# Oral health risks in adults who use electronic nicotine delivery systems and oral nicotine pouches: a critical review of the literature and qualitative synthesis of the available evidence

**DOI:** 10.1186/s12954-024-01147-y

**Published:** 2024-12-31

**Authors:** Gerhard Scherer, Nikola Pluym, Max Scherer

**Affiliations:** ABF Analytisch-Biologisches Forschungslabor GmbH, Semmelweisstr. 5, 82152 Planegg, Germany

**Keywords:** Combustible cigarettes, Electronic cigarettes, Heated tobacco products, Oral nicotine pouches, Oral health

## Abstract

**Background:**

Use of combustible cigarettes (CCs) and smokeless oral tobacco products are well documented risk factors for a variety of oral diseases. However, the potential oral health risks of using recently introduced (since about 2000) non-combustible tobacco/nicotine products (NCPs: electronic cigarettes (ECs), heated tobacco products (HTPs) and oral nicotine pouches (ONPs), remain poorly established.

**Methods:**

This review evaluates published human studies on detrimental oral health effects in people who use NCPs compared to those smoking cigarettes and those not using any tobacco/nicotine product (NU). We identified 52 studies, predominantly focusing on adults who used electronic cigarettes as an NCP. The studies exhibited significant heterogeneity regarding design, populations, endpoints and quality. Reported outcomes, based on both single and grouped endpoints were qualitatively evaluated by comparing people who use NCPs with NU and with people smoking CCs. Significant increases (indicating a worsening in oral health), significant decreases (indicating a lower level of detrimental effects) and no significant difference between groups were assigned scores of + 1, -1 and 0, respectively. Scores from studies belonging to the same single or grouped endpoints were averaged to a summary score ranging from − 1 to + 1.

**Results:**

The qualitative meta-analysis revealed that comparisons of EC *versus* NU groups yielded mean scores of 0.29 for pre-cancerous lesions (*N* = 14 observations), 0.27 for inflammatory processes (*N* = 83), 0.43 for oral clinical parameters (*N* = 93) and 0.70 for shifts in the oral microbiome (*N* = 10). The corresponding values for the EC *versus* CC group comparisons amounted to -0.33 (*N* = 15), -0.14 (*N* = 76), -0.27 (*N* = 78) and 0.57 (*N* = 7). Most studies had significant limitations regarding group sizes, duration of NCP use (mostly only a few years) and validity of self-reported exclusive NCP use. Notably, the implications of dual use (EC + CC) and prior CC use were often not adequately considered.

**Conclusions:**

The evaluated studies suggest that use of ECs is associated with relatively fewer detrimental oral health effects compared to smoking, yet oral health status remains poorer compared to not using any tobacco/nicotine products. These results have to be interpreted with caution due to a number of limitations and uncertainties in the underlying studies, particularly the potential biases and confounding factors inherent in cross-sectional study designs.

**Supplementary Information:**

The online version contains supplementary material available at 10.1186/s12954-024-01147-y.

## Introduction

Non-combustible nicotine/tobacco products (NCPs), such as e-cigarettes (ECs), heated tobacco products (HTPs), oral nicotine pouches (ONPs) and Swedish snus, have gained popularity as alternatives to combustible cigarettes (CCs) due to the perception of reduced harm [1]. In contrast to ECs, HTPs and ONPs, snus has a long history of use particularly in Sweden. Its chemical and biological properties, as compared to other oral tobacco types, allow snus justifiably to be regarded as a tobacco harm reduction product [2]. This review focuses specifically on the recent NCPs such as ECs, HTPs, and ONPs.

The oral cavity is the first organ affected by all tobacco and nicotine use forms, especially oral products like snus and nicotine pouches, which are in contact with the oral mucosa for up to several hours per day. However, also the use of inhalable products such as CCs, ECs and HTPs implies a direct contact of the released aerosols with the oral epithelial cells for a considerable time span.

The use of conventional tobacco products including CCs and various forms of oral tobacco is an established risk factor for oral cancer [3–5] as well as a number of non-malignant disorders such as leukoplakia [6–8], gingivitis [9, 10], periodontitis [11, 12], salivary gland function [13] and tooth damage [14, 15], delayed wound healing [16], bad breath (halitosis) [17], and dental staining [18]. NCPs deliver similar or somewhat reduced amounts of nicotine [19], but significantly lower amounts of toxicants [1, 20]. Use of NCPs was shown to be implicated with substantial reductions in the exposure to all classes of toxicants including aldehydes, epoxides, tobacco-specific nitrosamines (TSNAs), polycyclic aromatic hydrocarbons (PAHs), aromatic amines compared to use of CCs. This has been verified by measuring suitable biomarkers of exposure [21–24] (for review, see: [20, 25–28]).

Despite the considerable reduction (80–95%) in the exposure to tobacco combustion chemicals, NCP use still involves daily exposure to nicotine, matrix components, flavoring agents, and trace amounts of toxicants [20]. A systematic biomarker of exposure (BOE) study under controlled conditions with volunteers who use CCs, ECs, HTPs, oral tobacco (OT) and nicotine gum in comparison to NU revealed that persons who use OT (various products, not only snus) showed elevations in the exposure to TSNAs lower or close to that in participants using CCs [21–24]. There was some weak evidence that the exposure of individuals using HTPs to acrolein, acrylamide, acrylonitrile, o-toluidine and TSNA was slightly (but not significantly) higher than that of NU and the other non-CC groups, but much lower than that of individuals using CCs. Analytical data of product releases suggest that persons using ECs and HTPs might experience slightly elevated exposures to formaldehyde and acetaldehyde. However, there is no BOE-based support for this, due to lack of suitable BOEs. Persons using ECs are exposed to 1,2-propylene and glycerol in the upper mg range per day [29, 30].

These considerations on NCP use suggest that the exposure to toxic chemicals is low to negligible with the exception of nicotine, 1,2-propylene glycol and glycerol as well as some flavors. However, the frequent and long-lasting contact of the oral mucosa with low amounts of toxicants and presumably toxicological inert chemicals might have detrimental effects, including physical irritation, allergic reaction, drying or dehydration, disruption of the oral microbiome, and local pH changes [31–33].

Therefore, despite the substantial reduction in toxicant exposure compared to conventional tobacco products, it is crucial to investigate the potential adverse oral health effects of long-term NCP use. In a recent review of our group [34], the present knowledge of the overall health risks (including cancer, cardiovascular and respiratory diseases, oral cavity disorders, general oxidative stress and inflammation, reproduction, metabolic syndrome, and several others) and the particular role of nicotine in these disorders was summarized. The purpose of this review is to elucidate in more detail the reported effects of NCP use (ECs, HTPs and ONPs) on the oral mucosa in comparison to NU and individuals smoking cigarettes. We will focus on the following categories of biological endpoints:

- Oral cancer and pre-cancerous lesions, including DNA adducts in oral mucosa cells.
- Periodontitis and gingivitis as well as inflammation markers and other biomarkers of effect in oral mucosa cells.
- Changes in clinical markers of oral cavity, gum and teeth distortions.
- Shifts in the oral microbiome.

Ideally, these endpoints would be studied in persons using NCPs over mid- to long-term in comparison to NU and/or persons using conventional tobacco products (for the main part combustible cigarettes, CCs). However, due to the relatively short market presence of the NCPs of interest (< 20 years), there are as yet no long-term studies available which would allow to investigate outcomes such as cancer. Furthermore, the expectable heterogeneity of the available studies in terms of study design, populations, products and endpoints precludes a traditional (quantitative) meta-analysis. Therefore, we have opted for a qualitative synthesis of outcome data, aggregating various biological and clinical endpoints into the four categories listed above. This approach, while not a systematic review or meta-analysis, provides a clear and concise overview of the current state of knowledge. We acknowledge the limitations of this approach but believe it is justified given, the current evidence landscape, which will be discussed in detail later.

## Methods

This review was conducted according to the guidelines of PRISMA (Preferred Reporting Items for Systemic reviews and Meta-Analyses) [35], with the restriction explained in the previous section.

### Libraries, search strategy, inclusion and exclusion criteria

**Fig. 1:**
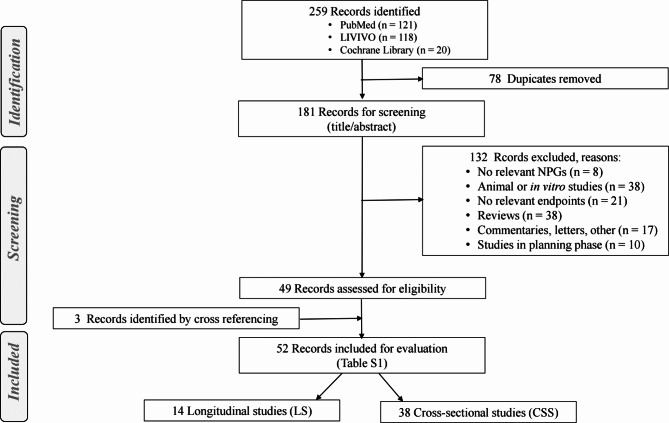
Flow chart for identification, excluding and including studies for evaluation in this review according to PRISMA [35]

The online literature databases PubMed, LIVIVO and Cochrane Library were searched for the major topics NCPs of interest (ECs, HTPs, ONPs) and oral health disorders with simultaneous application of filters for human studies and the languages English or German. The orignial search in the three databases was performed on November 9, 2023, updates for recent publications were made until August 2024. Since this is a rapidly evolving field, some relevant papers may have been missed due to the delay between the search and the completion of the review. The number of hits were in total 259, with 121, 118 and 20 obtained from PubMed, LIVIVO and Cochrane Library, respectively. After removing 78 duplicates, 181 articles remained, of which the titles and abstracts were screened for meeting the inclusion and exclusion criteria. Inclusion criteria comprised human studies with individuals who use NCPs. Snus and any other tobacco products were not explicitly included in the search strategy, because this would go beyond the boundaries of the review. A separate comparison of oral health effects of snus with ONP would be worthwhile at a later point in time, when more suitable studies with ONPs will be available. Observed effects or outcomes need to be compared to NU (negative controls) and/or persons using CCs (positive controls). Study endpoints must be any oral health effects, including cancer, pre-cancerous lesions (including cytogenetic effects and DNA adducts), inflammatory processes (including changes in pro- or anti-inflammation biomarkers in oral tissues, GCF, saliva or other oral fluids), dental issues, any changes in clininal oral health parameters such as BOP, CAL, PD, PI, PS, MBL, PIBL. Exclusion criteria comprised animal, in vitro and clinical case studies, reviews, commentaries and letters as well as studies in the planning phase. Application of these inclusion and exclusion criteria resulted in 49 articles for evalution. Cross referencing from recent reviews and meta-analyses on NCPs and oral health revealed additonal 3 studies suitable for this review so that a total of 52 studies were included in the final evaluation, 14 of the longitudinal study and 38 of the cross-sectional study type (Fig. 1).

### Information extracted from the included studies

The information of the included studies was extracted according to a standardized procedure and presented in Table S1 (Supplemental file). The NCP(s) investigated (ECs, HTPs, ONPs) together with negative controls (NU or persons who do not smoke (NS), if so defined in the study) and positive controls (usually individuals using CCs) are shown in column 2 of Table S1. Terminology and definitions for tobacco/nicotine product use (or non-use) are in general those used in the original articles with some adaptions. For general definitions of these groups, see the list of abbreviations for DU, FS, NS, NU and NV. Study type, study groups with group sizes as well as mean age and gender of the persons are provided in column 3. The history of tobacco/nicotine product use of the investigated study groups is summarized in column 4. The extracted information on use history comprises how the product use was assessed (self-reports, questionnaires) and whether or not the exclusive NCP use was verified (e.g. with suitable biomarkers of exposure). Endpoints and outcomes (if possible in quantitative terms) together with statistical significances for the differerences between groups are shown in column 5. The studies were assigned to four major outcome groups: (i) pre-cancerous lesions in oral cells, fluids and tissues, including cytogenetic changes (e.g. micronuclei), DNA adducts and oxidative stress markers; (ii) inflammatory processes and changes in related biomarkers of biological effects; (iii) changes in clinical parameters of the oral cavity including teeth; (iv) shifts in the oral microbiome in various oral fluids. In the last column of Table S1, comments are provided, mainly on the strengths and weaknesses of the study.

For this review on detrimental oral health effects in persons using NCPs compared to NU and persons using cigarettes (CC), 52 human studies, 38 cross-sectional and 14 longitudinal studies fulfilled the inclusion criteria as described in Sect. 2.1. The information extracted from these studies is shown in Table S1. Study sizes were highly variable and comprised between 30 and 1 million persons with most studies encompassing 60–120 persons. Participants, in general, were healthy adults, but some studies included patients with oral health problems such as periodontitis or caries. Age of participants covered a range of 20–80 years with a focus on young to middle ages (25–50 years). Most studies comprise both sexes, while a few included only males.

In the selected 52 studies, almost only the effects of EC use were investigated. In two studies [36, 37] ONPs and in one study [23] HTPs were investigated. A recent update of the literature research revealed two additional studies [38, 39] on oral health effects of HTPs. In the study synthesis only EC studies were included. A differentiation between EC types, generations, nicotine content, and added flavors was not considered in the analysis, primarily to avoid too small group sizes.

### Synthesis of the reported results from various studies

For synthesis of the extracted results from the included studies (Table S1), the reported findings were transformed to ‘qualitative’ categories (-1, 0, + 1, as defined below). The rationale for this approach is the fact that a large number of endpoints (*N* = 68) have to be evaluated, with 15, 24, 26 and 3 different endpoints for the categories pre-cancerous lesions (i), inflammatory processes (ii), clinical parameters for oral disturbances (iii) and shifts in the microbiome (iv), respectively. Furthermore, the data extracted from the included studies entail a high degree of heterogeneity in terms of study types and group sizes, persons (gender, age), product properties, clinical and analytical methodologies applied and clinical/biological endpoints measured, which precludes the application of classical (quantitative) meta-analyses [40, 41]. Of major interest in this review were significant differences in endpoints or categories of endpoints between groups, namely persons who use NCPs *versus* NU and *versus* smoking. Outcomes of cross-sectional studies as well as baseline results of longitudinal studies were represented as statistical significantly (*p* < 0.05 or better) increased effect (score: +1), significantly decreased effect (-1) or no (significant) difference (0). The scores + 1 or -1 for the various endpoints were assigned to the effect: worsening (+ 1) or a change for the better (-1). Outcomes for single endpoints as well as the groupwise (i – iv) synthesis are shown in Table S2. Summary scores for endpoint groups (i – iv) were calculated as means with 95% confidence intervals (CI). If 10 or more observations for single endpoints were available, means and 95% CIs were also calculated for these. This was the case for the endpoints TNF-∝, IL-1ß, IL-6, PI, BOP, PD and MBL (Table S2). Baseline results in longitudinal studies were treated simialr to data from cross-sectional studies. Differences over time (baseline *versus* follow-up (FU)) were originally planed to be evaluated as changes within (*intra*) or between groups (*inter*). However, reported results of longitudinal studies were too heterogeneous so that evaluation across studies was not meaningful. Time trends of oral effects in a few interesting longitudinal studies are presented in a ‘narrative’ approach.

## Results

### General study characteristics

History of tobacco/nicotine products use was of particular impotance for evaluation of the oral health risks associated with NCP use. Most studies rely on self-reports assessed with questionnaires (Table S1). In 11 studies [42–51], cotinine or other nicotine metabolites were determined in body fluids (blood, saliva, urine), which allows for the distinction between persons who use any tobacco/nicotine product (including NCPs) and NU as well as the extent of product use. However, it does not distinguish between NCP and CC use. This is possible, at least in terms of short-term use, with the biomarkers carboxyhemoglobin (COHb) and exhaled carbon monoxide (COex), which had been applied in 10 studies [43, 46, 48–50, 52–55]. A longer period of CC use *versus* NCP use is assessable by NNAL in urine and was reported in one study [52]. Urinary CEMA, a biomarker of exposure to the combustion product acrylonitrile, was determined in three studies [23, 43, 52].

Duration of NCP use was reported in only part of the selected studies. In 4 investigations [56–59], NCP (almost exclusively EC) use of at least for 1 year was required for study participation. In another 5 studies [44, 47, 60–62], mean NCP use durations between 2 and 3 years were reported. The longest average NCP use durations in the selected studies amounted to 6.4 [63], 9.2 [64], 12.2 [65] and 12.5 years [66]. One study [53] provided a mean use time for ECs of 21.6 years, which probably is an error, given the fact that ECs are on the market since about 2007 and the mean age of persons using ECs in that study was reported to be 41.5 years.

Dual use (mostly EC together with CC) is heterogeneously wielded in the selected studies. In 8 studies [47, 51, 67–72], persons reported to use both CCs and ECs concurrently (DU) were assigned to a separate group or conciously included in a special NCP group. In 12 studies [42, 59–61, 66, 73–78], it was stated that DU were excluded. The remaining studies did not mention or consider the issue of dual or multi-product use, although it is likely that it occured in the respective investigations. Our evaluation is based on persons using exclusivly NCPs, as far as this is possible with the study information provided. Unassessed dual use can significantly bias and confound the estimated oral health risks of NCP use (discussed later).

The evaluation of oral health risks in persons using NCPs comprised 65 different endpoints (Table S2), which were assigned to 4 groups (defined in Sectin 2.2). In total, 199 single observations were extracted and evaluated from the 52 selected studies.

### Pre-cancerous endpoints

**Fig. 2:**
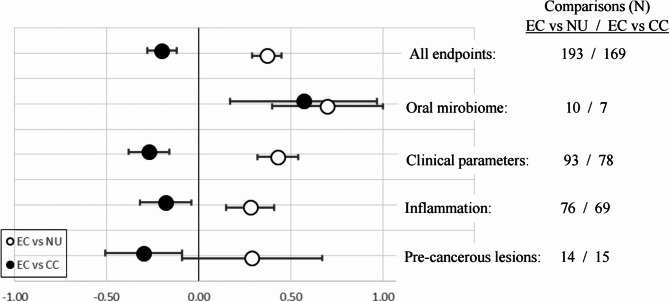
Mean scores with 95% confidence interval (CI) for all endpoints and the 4 endpoint categories extracted from the 52 studies selected for this review. Open circles (○) : comparison between EC und NU groups, filled circels (●): comparison between EC and CC groups

In total, 12 different endpoints could be extracted from 12 studies [23, 43, 47, 49–51, 53, 56, 59, 65, 77, 79] with 16 single observations (Table S2). Only the endpoints ‘micronulei’ [53, 77, 79] and ‘NNN in saliva’ [23, 65] were investigated in more than one study. Oral leukoplakia, a frequent lesion in persons using CCs [6–8], was reported in only one of 45 persons using ECs in one study [80]. All other endpoints were determined in only one investigation. There were 14 EC *versus* NU and 15 EC *versus* CC group comparisons included in the qualitative analysis, which yielded mean scores of 0.29 (95% CI: -0.09–0.67) and − 0.33 (-0.58 - -0.09), respectively. The results are graphically depicted in Fig. 2. In one investigation [23], the endpoint ‘NNN in saliva’ was compared in persons who used HTPs to NU and to persons using CCs, resulting in scores of 1 and 0, respectively.

Only recently, Majid [81] observed changes in salivary lipid profile and oxicative stress markers in young adults who smoked (CC) and adults using ECs, which the authors assumed to represent a hidden threat to oral health.

### Inflammatory processes

This group of effects comprised 21 different endpoints derived from 19 studies [36, 44–46, 48, 51, 57–60, 66, 69, 70, 72–74, 82–84] with 83 single observations (Table S2). The most frequently determined endpoints were IL-1ß (12 observations), IL-6 (10 observations) and TNF-∝ (10 observations). A qualitative analysis for markers with at least 10 observations including the inflammation biomarkers TNF-∝, IL-6 and IL-1ß, is provided in Sect. 3.7. There were 76 comparisons between EC and NU groups resulting in a synthesized mean score for inflammatory processes of 0.28 (95% CI: 0.15–0.41) and 69 EC *versus* CC group comparisons with a mean score of -0.16 (95%-CI: -0.28 - -0.04) (Fig. 2). Note that due to their anti-inflammatory properties, the marker IL-8, IL-9, IL-10, IL-13 and IL-RA were inversed (i.e., the algebraic score signs +/- were inverted).

In a recent publication of 2024, Garcia et al. [85] reported significantly increased risk of gingivitis and white spot caries lesions in vaping students compared to NU. In those students reporting also use of CCs (in addition to EC use), significant increases compared to NU were observed in gingivitis (borderline), caries, white spot caries and nicotine stomatitis. Comparisons between the CC group and NU revealed the same differences as found in the DU *versus* NU comparison, but also increased tooth loss and dentinoenamel staining in the CC group.

### Clinical parameters for oral disorders

This group of effects comprised 25 different endpoints derived from 20 studies [42, 44, 46, 56, 57, 59–64, 66–68, 71, 73–76, 78, 80, 82, 83, 86–90] with 97 single observations (Table S2). The most frequently determined endpoints were PD (15 observations), PI or PS (14 observations), BOP (13 observations), and MBL (11 observations). A qualitative analysis for markers with at least 10 observations, including the clinical biomarkers MBL, BOP, PI/PS, and PD, is provided in Sect. 3.7. There were 93 EC *versus* NU group comparisons resulting in a synthesized mean score for clinical parameters of oral distortions of 0.43 (95% CI: 0.32–0.54) and 78 EC *versus* CC group comparisons with a mean score of -0.27 (95% CI: -0.38 - -0.16) (Fig. 2). Note that score inversions were performed for IgA, lysozyme, lactoferrin and BOP.

In a recent cross-sectional study, La Rosa et al. [39] found that persons who use ENDS (including ECs and HTPs, all formerly smoked but switched at least 6 months ago, COex: <7ppm) exhibited reduced accumulation of dental plaque and calculus compared to persons currently smoking. Extent of dental plaque and calculus was similar to that of persons who never (< 100 CC in their life) or formerly smoked (> 6 months ago, COex: <7ppm).

A study on dental coloring [38] found that exclusive use of ECs and HTPs (both groups formerly smoked, > 6 months ago, COex: <7ppm) were associated with less dental coloring than in persons currently smoking (10 + cigarettes/d, COex: ≥7ppm).

### Shifts in the oral microbiome

This group of effects comprised 4 different endpoints derived from 7 studies with 10 single observations (Table S2). Reported changes in the oral microbiome in people using ECs compared to NU include significantly higher alpha-diversity [45] and significant differences in beta-diversity [91, 92], indicating overall compositional changes in the oral microbiome by vaping. Elevated levels in persons using ECs in comparison to NU were found for Veillonella [91, 92], Haemophious [91] as well as (at the subgingival site) increases in Actinomyces, Rothia Neisseria and Enterococcus [45]. These changes suggest an increased risk for oral inflammation and infection in persons using ECs compared to NU.

In the qualitative analysis for shifts in the oral microbiome, no differentiation between favorable (-1) or unfavarable (+ 1) changes were made. Rather, all significant shifts between groups were assigned with a score of + 1. There were 10 EC *versus* NU group comparisons resulting in a synthesized mean score for shifts in the oral microbiome of 0.70 (95% CI: 0.40–1.00) and 7 EC *versus* CC group comparisons with a mean score of 0.57 (95% CI: 0.53–0.97) (Fig. 2).

### All oral endpoints

In total, 65 different oral endpoints from 52 studies with 199 single observations were assessed (Table S2). For the qualitative meta-analysis (Sect. 2.3), there were 193 data sets with EC *versus* NU group comparisons resulting in a mean score for oral distortions of 0.37 (95% CI: 0.29–0.44) and 169 EC *versus* CC group comparisons with a mean score of -0.19 (95% CI: -0.26 – -0.11) (Fig. 2).

### Qualitative meta-analysis for the most frequently applied single endpoints

**Fig. 3:**
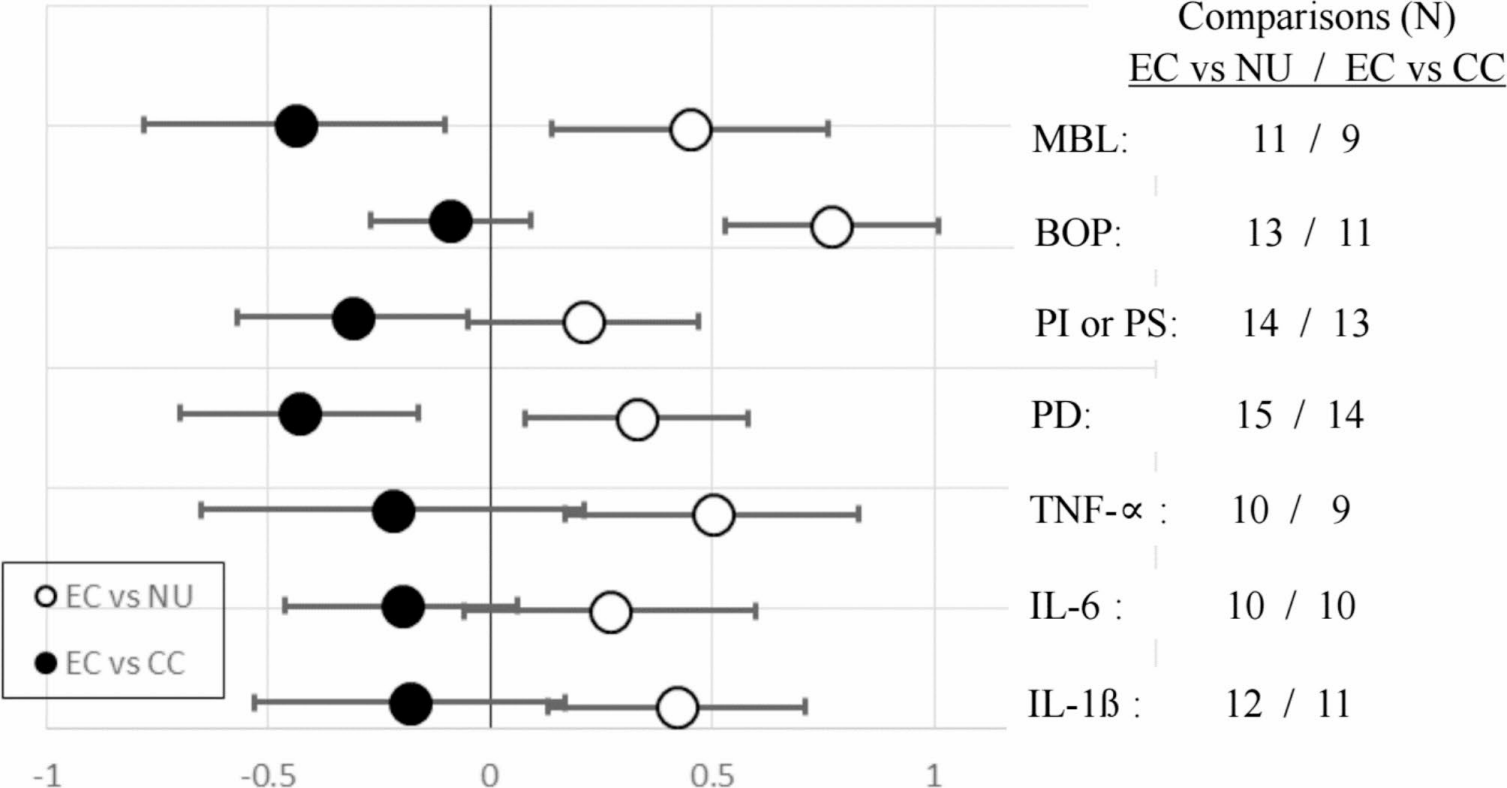
Mean scores with 95% confidence interval for single endpoints of detrimental oral effects reported in 10 or more data sets. Open circles (○): comparison between EC und NU groups, filled circles (●): comparison between EC and CC groups

For single endpoints applied in 10 or more different studies, analyses were conducted as described in Sect. 2.3. This was the case for 7 out of 65 endpoints, namely IL-1ß, IL-6, TNF-∝, PI or PS, BOP, PD and MBL. Results of this analysis are shown in Fig. 3.

It is obvious that CI ranges in general were larger than in Fig. 2, which primarily is caused by the lower number of observations for the single endpoints. It is interesting to note that BOP (for which the score was inverted) showed the highest mean score of all EC *versus* NU comparisons (0.77) and the lowest of all EC *versus* CC comparisons (-0.09), indicating that BOP in individuals using ECs was most likely different from NU, but not different from the CC group. The reason for this finding is discussed later.

### Outcomes of longitudinal studies

The selected studies on oral health effects in individuals who use NCPs comprise 14 longitudinal studies [23, 37, 43, 48, 52, 55, 56, 58, 60, 84, 86, 87, 91, 93] (Table S1). Baseline (BL) data from these longitudinal studies were treated as cross-sectional studies and included in the results presented in Sect. 3.2 to 3.7. Longitudinal data, which would allow the analysis of changes over time (in longitudinal studies usually at BL and one or more follow-ups (FU) time points were analyzed). Longitudinal study results are regarded as superior to those obtained from cross-sectional studies (for a number of reasons, discussed below), provided that the covered time periods (baseline to follow-up) were sufficiently long [94, 95]. For obvious reasons this was not the case in the longitudinal studies selectable for this review. The time periods covered a range of from 3 days to about 2 years, with the highest frequency for follow-ups at 6 months. In general, longitudinal studies had smaller group sizes and were more heterogeneous than cross-sectional studies, thus preventing a synthesized analysis (meta-analysis) across studies. Therefore, changes over time of oral effects reported in single longitudinal studies were briefly presented in the following.

In a longitudinal study with FUs at 2, 4 and 6 weeks, a normalization of reversible histological changes were observed in 60 individuals who used snus after replacing snus with ONPs already at the first FU visit [37]. Unfortunately, no negative control group (using no nicotine/tobacco product during the FU period) or positive control group (continued use of snus) was included.

In a longitudinal study with 30 persons using ECs and 30 persons using CCs, no differences in the oral clinical parameters CAL, PD, MBL and PS were found at baseline, while after 6 months follow-up, PD, CAL and MBL became worse in the CC compared to the EC group [56]. Both groups showed significant correlations between the long-term dose and the endpoints MMP-8, CTX, PD, and CAL. This study did not contain a negative control group (NU).

In individuals with moderate chronic periodontitis under SRP treatment (36 persons using ECs and 35 NU), PI, PD, MBL, CAL, IL-4, IL-9, IL-10, and IL-13 (the latter 4 in GCF) were reported not to be significantly different at baseline [58]. In the EC group at 3 months follow-up, the endpoints PI, GI, PD, CAL, and MBL were found not to be different from baseline, while PI, GI and PD were significantly reduced in NU. For the anti-inflammation markers IL-4, IL-9, IL-10, and IL-13, a significantly higher increase was observed in the non-smoking compared to the EC group. Vaping apparently mitigated the SRP treatment effect.

In another longitudinal study with 89 male FMUS patients (30 who use CCs, 28 who use ECs, 31 NU), improvements in PI and PD were observed after 3 as well as 6 months follow-up in all three groups. The differences were significant between baseline and 3 months later in all groups. Extent of improvements was in the order NU > EC > CC [60].

In an intervention study with 80 persons using CCs suffering from periodontitis, 40 switched to EC and 40 stopped smoking [93]. At 6 months follow-up, PD improved to a similar extent in both groups. Oral dryness, however, did not change in individuals using ECs, while it decreased in NU.

Tatullo et al. [55] investigated the clinical parameter PI and PBI in 60 persons using ECs with ≤ 10 years of previous smoking (CC) compared to 50 vaping persons with > 10 years previous smoking. Measurements at baseline, 60 and 120 days later revealed improvements over time in both groups. However, in the group with the longer period of former CC use, the oral clinical parameters were less advantageous than in the comparator group.

A cohort study over 6 months with 27 individuals using CCs, 28 using ECs and 29 NU revealed that the ∝-diversity (a measure of microbiome diversity applicable to a single sample) increased across the cohorts longitudinally, yet each cohort maintained a unique microbiome [48]. The authors concluded that EC use promoted a unique periodontal microbiome (as a stable state between smoking and NU), presenting an oral health challenge.

In a similar study design, Xu et al. [84] investigated the salivary microbial composition in 101 patients with periodontitis (group sizes: 31 CC, 32 EC, 38 NU). From their results, the authors concluded that vaping, similar to smoking, alters the bacterial composition with an increase of disease-associated pathogens.

Similar observations were reported by Chopyk et al. [91]. Furthermore, it was reported that reducing the EC use over 2 weeks led to a decrease of pathological changes in the salivary but not in the buccal microbiome.

## Discussion

### General considerations

For this review on oral health effects of NCPs (scheduled to comprise ECs, HTPs and ONPs), 52 human studies (38 of cross-sectional and 14 of longitudinal type) were selected and subjected to a series of qualitative meta-analyses. It turned out that in only 3 studies, NCPs other than ECs were investigated so that the analyses only dealt with the use of ECs (vaping) and refer to comparisons of EC groups *versus* NU and EC *versus* CC groups. Classical (quantitative) meta-analysis for disease endpoints, for example oral cancer, are presently not possible for a number of reasons. A foremost reason is the fact that duration of NCP use was not sufficiently long to induce chronic diseases (cancer would require more than 2 decades). ECs, also known as ENDS (electronic nicotine delivery systems), in its modern form have been invented in 2003 by the Chinese pharmacist Hon Lik and were first introduced to the market in about 2007 [96]. HTPs (with electric heating systems) have been marketed from the end of the 1990’s. ONPs are the most recent products of the NCPs dealt with in this review. The big tobacco companies started marketing the products as tobacco-free ONPs in 2019 [97]. ECs, which were in the focus of this review, were subject to rapid product changes so that since its general marketing at least four generations were passed through [98, 99]. This factor can significantly contribute to the heterogeneity of the study data to be evaluated. Other factors include study type, group sizes, gender, age as well as endpoints investigated and methods for their determination. Under these premises, we decided to perform qualitative syntheses for single endpoints with at least 10 observations in different studies or groups of endpoints belonging to four different types of oral disorders, namely (i) pre-cancerous lesions including oxidative stress markers, (ii) inflammatory processes, (iii) general clinical parameters used for oral (including dental) disorders, and (iv) shifts in the oral microbiome. Assignment to these endpoint categories might appear somewhat arbitrary, however, there is multiple evidence that many, if not all, oral endpoints considered in the this review are mechanistically connected in the development of various oral disease [94, 95, 100–103]. We, therefore, believe that our approach of combining different endpoints to groups of disorders or even a total score for detrimental effects in the oral cavity is physiologically justified for the purpose of qualitative meta-analyses. The procedure for this analysis is described in detail in Sect. 2.3. In essence, the study findings with respect to the EC *versus* NU and the EC *versus* CC group comparisons for each endpoint were categorized to three ‘scores’, namely ‘+1’ (= significant increase (in the sense of a worsening or unfavorable change) of an oral health condition), ‘-1’ (= significant decrease (in the sense of an improvement) of an oral health condition) and ‘0’ (= no significant difference between groups). It has to be noted that for some endpoints an increase can imply a favorable change for oral health. This was considered accordingly in the analysis (Table S2). Furthermore, it has to be emphasized that the calculated means and 95% confidence intervals (CI) represent probabilities for finding an unfavorable (positive score values) or a favorable change (negative score values) for individuals using ECs compared to either NU or persons smoking CCs.

From Figs. 2 and 3, it is immediately evident that using ECs was associated with more unfavorable oral health outcomes compared to NU. Whereas people using ECs were generally reported to do better in terms of oral health compared to those using CCs. This is in general agreement with all reviews on this topic published in the last 5 years [94, 95, 99–121]. However, means of summary scores in Figs. 2 and 3 were most frequently between 0 and + 0.5 (EC *versus* NU group comparisons) or between − 0.5 and 0 (EC *versus* CC group comparisons), indicating that the outcomes of the evaluated studies were not very consistent. The higher mean scores ( > + 0.5) calculated for the oral microbiome (Fig. 1) and BOP (Fig. 3) are discussed below.

In general, smaller numbers of observations were available for the evaluation of the 7 single endpoints (Fig. 3), leading to larger CI ranges. Of the 14 comparisons, 5 CIs included zero, suggesting considerable inconsistencies in the outcomes for these endpoints.

### Oral cancer

As yet, no epidemiological studies on the oral cancer risk of chronic NCP use are available. As a surrogate, the association between NCP use (usually of moderate duration only) and various pre-cancerous lesions mechanistically related to oral cancer was evaluated in our review (Sect. 3.1) as well as in a number of previously published reviews [95, 100, 101, 112, 119, 120]. All evaluations (including our own) came to the conclusion that the available evidence suggests that EC use may have the potential to increase the oral cancer risk by working through one or several possible mechanisms which might include formation DNA adducts by exposure to potentially genotoxic chemicals in EC aerosol (e.g. acrolein, NNN, others), DNA damage (e.g. indicated by the increased formation of micronuclei), oxidative stress, suppression of the immune system, and shifts in the oral microbiome. There is also general agreement in the literature that conclusive answers with regard to the role of chronic vaping in oral cancer induction would require about another decade. The evaluation of an involvement of the habitual use of the other two NCPs of interest (HTPs, ONPs) in oral cancer generation is not possible, due to the lack of suitable data.

### Oral inflammations

A large number (*N* = 24) of different endpoints on inflammatory processes were available from 20 human studies, allowing 76 EC *versus* NU and 69 EC *versus* CC group comparisons (Sect. 3.2, Fig. 2). In addition, qualitative meta-analyses were also conducted for the single inflammation biomarkers IL-ß, IL-6 and TNF-∝ (Fig. 3). Although the results are partly inconsistent, the majority of studies reported elevated inflammation in EC groups compared to NU and decreased occurrence of inflammation in EC compared to CC groups. This is in general agreement with other surveys of the literature [94, 108, 109, 114–116]. There is no established knowledge about the mechanism of how and by which chemicals vaping can induce inflammatory processes in the oral cavity. Assumptions include the involvement of nicotine [108], metals [94] or shifts in the oral microbiome caused by dry mouth and reduced salivary flow [109] or increased growth of *Candida albicans* [102].

No studies on inflammation effects of HTP or ONP use were identified for this review.

### General clinical endpoints for oral disorders

The general clinical parameters for oral disorders comprised 25 different endpoints extracted from 20 human studies, allowing 93 EC *versus* NU and 78 EC *versus* CC group comparisons (Sect. 3.2, Fig. 2). In addition to the group evaluation, qualitative meta-analyses for the single endpoints PD, PI or PS, BOP and MBL were also conducted (Fig. 3). The majority of studies showed significant increases in clinical disorders in EC compared to NU groups and significant decreases in clinical disorders in EC compared to CC groups. The ranking for groups showing clinical disorders (NU < EC < CC) was also reported in other recent reviews on detrimental oral effects of vaping [102, 114, 116, 120]. With one exception (BOP), the responsible chemicals in EC aerosol and the mechanisms for the reported clinical effects are not established. As possible candidates nicotine [114, 116], metals [114, 116], flavor components [102, 114, 116], sucrose and sugar substitutes [102], as well as acids [102] were discussed. BOP was found to be reduced in persons using ECs compared to NU and very similar to that observed in persons using CCs (Fig. 3). This consistent finding can be explained by a nicotine-caused vasoconstriction in gum tissue (for review see [34]). A similar effect on BOP has to be expected in people using HTP and ONPs. However, no investigations on BOP and any other detrimental oral effects of these products were available for this review.

### Oral microbiome

Four endpoints on the oral microbiome were selected from 7 studies for evaluation. This data set al.lowed 10 EC *versus* NU and 7 EC v*ersus* CC group comparisons. In contrast to the evaluation of the other endpoints or groups of endpoints, qualitative meta-analysis was modified in so far as differences (shifts) between groups in the oral microbiome were not divided into significantly unfavorable (+ 1) or favorable changes (-1), rather, any significant shift was assigned the score value of + 1 (unfavorable). The reason for this approach was that the impact of a shift in terms of increasing or decreasing an oral health risk cannot yet be predicted with sufficient certainty. It is, therefore, assumed that significant changes in the oral microbiome represents a potential oral health risk. Figure 2 shows that in most studies vaping was found to lead to a shift in the composition of the oral microbiome, both compared to NU and smoking (CC). This is in agreement with most results from other recent reviews [99, 106, 108–110, 113, 115, 120]. The mechanism by which vaping can change the oral microbiome is not yet well established. An interesting hypothesis assumes that use of ECs can lead to dry mouth (xerostomia) by action of PG and VG (which are hygroscopic) together with an increase in biofilm volume and a reduced salivary flow [109, 110]. A role of nicotine in oral microbiome change has not yet been reported [34], which accords with another recent review, stating that nicotine may not be involved in oral microbiome shifts [108]. The oral health consequences of shifts in the oral microbiome are as yet not quite clear [108]. Other authors speculated that a chronic change in the oral microbiome can lead to severe health disorders such as periodontitis and periodontal disease and other oral health issues [109, 110, 113, 115], retarded wound healing [109] and even increased oral cancer risk [109].

### Role of major constituents in the EC aerosol (vapor)

All e-liquids and hence the EC aerosol inhaled by a person contain glycerol (usually vegetable-derived glycerol, VG), 1,2-propylene glycol (PG), nicotine and flavor compounds, although in varying ratios and amounts. In particular the added flavors may differ largely in composition as well as quality and quantity of components from brand to brand.

The e-liquid matrix usually consists of VG and PG in varying ratios. The mouth-level exposure to these compounds is in the gram per day range [20]. It is, therefore, not unreasonable to assume that vaping may lead to changes in the oral biofilm and shifts in the composition of the microbiome, connected with xerostomia [70, 109–111].

Guo et al. [52] found in vitro evidence that PG can inhibit bacterial inflammation and the formation of AP sites in DNA, thus explaining their finding that persons who vape have significantly lower AP levels in oral cells than those using CCs and those using no tobacco/nicotine products.

The nicotine content in e-liquid most commonly ranges from 3 to 36 mg/mL, with an upper limit set for the European Union of 20 mg/ml [122]. The mean daily intake of nicotine by vaping was estimated to be about 10 mg/d [20]. A recent review on the role of nicotine in various oral disorders and diseases did not identify nicotine to be a major risk factor for oral diseases [20]. A consistent finding was that BOP decreases in individuals using any nicotine product compared to NU (Table S1 [94]), . Our analysis also confirms this nicotine-related effect, showing that EC and CC groups were comparable in their BOP level, but rather different from NU (Fig. 3). By a similar mechanism, nicotine can be involved in retarded wound healing processes in the mouth by inducing local ischemia [109].

Holliday et al. [111] concluded that salivary nicotine concentrations in persons using CCs and probably also in those using ECs (4–10 µM) are unlikely to exert cytotoxic effects in the oral cavity. In another review [112], it was hypothesized that nicotine in the oral cavity can cause a number of detrimental effects, mostly mediated via the nicotinic-acetylcholin-receptor (n-AChR), supportive in the development of oral diseases including oral cancer.

Information on detrimental effects in the oral cavity of flavors added to NCPs are sparse. In a review on detrimental oral health effects in persons who vape, Irusa et al. [123] focused on the effects of flavors. The authors stated that with certain flavors, investigators were able to show a 4-fold increase in microbial adhesion to enamel, a 2-fold increase in biofilm formation, and a 27% decrease in enamel hardness. From an evaluation of the literature, Flach et al. [95] came to the conclusion that clinical evidence urges to assume that flavored e-liquids appear to be more harmful. Ebersole et al. [108] pointed out that toxic agents can be released from flavor compounds in e-liquid upon heating.

In general, it has to be stated that the flavor compounds added to NCPs have GRAS (“generally recognized as safe”) status, meaning that they are safe to be used in food. An issue, however, could be the release of toxicants (mainly aldehydes) as mentioned above [108].

### Bias, confounding, and study limitations

It is assumed that the human studies evaluated for this review were subject to bias and confounding. Table S1 contains comments and statements with respect to bias and confounding for each of the 52 evaluated studies. For quality assessment of the included studies, the Newcastle-Ottawa-Scale (NOS) for observational studies of the case-control type can, with certain restraints, be applied [124]. The NOS defines three domains for quality assessment of case-control studies: (i) selection of cases and controls (in our review: persons who use NCPs, persons not using any tobacco/nicotine products (NU) and persons using cigarettes (CC), (ii) comparability of groups, and (iii) ascertainment of exposure. A fourth domain would have to be added: (iv) outcomes and endpoints, which were of particular importance in this review, as multiple endpoints (actually *N* = 65) were included in this analysis. In particular, endpoints applied here were less well estblished and pre-staged, rather than manifested diseases. As described in Table S1 and at various passages in the text, selected studies were highly heterogeneous in many study features, including the three domains of the NOS. However, we see especially high potential of bias in the domains (i) selection of individuals using ECs and (iii) ascertainment of exposure. With a few exemptions, there are weaknesses in almost all studies in Table S1. Given the fact that most individuals using ECs were switchers from CC, possible long-term ‘carry-over’ effects of former smoking have to be considered. For example, in a longitudinal study over 120 d it was shown that persons who currently vape and formerly used CC for > 10 years had a worse periodontal status than those with ≤ 10 years of former smoking [55]. In many of the selected studies, the former smoking status of the EC group was not or only insufficiently assessed. Furthermore, the assessment of EC use was most frequently performed by means of (structured or unstructured) questionnaires or interviews. In only a few studies, at least the short-term compliance of exclusive EC use was verified by suitable biomarkers such as COex or COHb, CEMA, NNAL [23, 48–50, 52, 55]. In none of the studies, a long-term biomarker for CC use was applied, such as for example the hemoglobin adduct CEVal (2-cyanoethyl valine, a long-term biomarker for exposure to acrylonitrile) [125]. Among adults (18 + years) who use ECs in the US in 2021 (about 11.6 million persons), almost 30% were DU (using CCs and ECs) [126]. It has to be assumed that it is quite likely that the EC groups in the evaluated studies contain considerable numbers of un-assessed DU, which might constitute a potential risk of bias for elevated deterimental oral health effects in the EC groups. The extent of this bias caused by DU and former smoking is difficult to quantify, but in all likehood part of the generally observed increase in detrimental oral health effects might be attributable to some current and former smoking (CC) in the EC groups.

A well known confounding factor in oral health effects is a lack in dental hygiene. Adults using CCs, on average, were found to practice lower oral hygiene than those not using cigarettes [127]. It is not yet known whether the same applies to persons using ECs compared to NU. In a very recent review, however, no such difference was reported [128].

### Limitations

Beyond the general risk of bias and confounding implicated with cross-sectional study designs described in the previous section, there is the unavoidable limitation that duration of use of NCPs is presently too short for the investigation of long-term detrimental effects such as oral cancer. As a substitute, precursor lesions or (early) biomarkers of biological effects (BOBEs) were used as endpoints for the evaluation of detrimental oral health effects in NCP using individuals. The predictive power of these biomarkers for subsequent diseases is as yet not well established. Another limitation was that many of the selectable studies have small group sizes (< 50 persons/group), which may prevent finding small differences between groups to be significant. This could have a direct impact on the qualitative meta-analyses conducted in this review.

The approach of a qualitative meta-analysis as introduced here is not an established methodology. Its limitations have been discussed earlier in the text. The calculated summary scores and CI-values may, by no means, be interpreted as oral health risks. Rather, the score values (range: -1 to + 1) indicate the probability of finding an unfavorable (+) or favorable difference (-) between two groups when a sufficient number of comparisons was performed. Another limitation of this approach is that the effect size as well as group sizes are not taken into account.

The combination of various endpoints to a new oral health parameter (as was done for the categories ‘pre-cancerous lesions’, ‘inflammatory processes’, ‘oral clinical parameters’, ‘shifts in the oral microbiome’, ‘total oral disorders’) can be criticized to be arbitrary and not (always) physiologically based. In general, we would agree with this criticism. However, we would argue that many (if not even all) of the endpoints were interrelated via one or more physiological pathways, so that even combining all detrimental oral endpoints to a variable ‘total oral disorders’ (Fig. 3) may have a certain justification.

## Conclusions

Our qualitative meta-analyses of a multitude of endpoints of early oral discorders revealed that individuals using ECs showed somewhat lower levels of detrimental effects in the oral cavity than persons using CCs, but still increased levels compared to NU. Systematic bias by presumably underreported dual use and also former smoking might be responsible for part of the oral disorders in the EC groups. This is of particular importance in cross-sectional studies. There is a need for further research into the long-term effects of NCP use on oral health outcomes, as well as the underlying biological mechanisms. Since ONPs have the most intensive contact with the oral mucosa, future studies on the oral health effects of this product are of particular importance.

## Electronic supplementary material

Below is the link to the electronic supplementary material.

Supplementary Material 1

Supplementary Material 2

## Data Availability

This paper only refers to published data appropriate citations are provided.Information on further details of the evaluation can be provided by the corresponding author upon request.

## Abbreviations

3-OH-Cot: Trans-3’-hydroxycotinine
3-OH-Cot-gluc: Trans-3’-hydroxycotinine-N, O-glucuronide
8-OH-dG: 8-Hydroxy-2’-deoxyguanosine (a BM of oxidative stress)
AP: Apurinic/apyrimidinic (sites in DNA)
B: Blood
BL: Baseline (at start of a longitudinal or prospective study)
BLT: Bone loss around teeth
BM: Biomarker
BOP: Bleeding on probing
CAL: Clinical attachment loss
CBL: Crestal bone loss
CC: Combustible cigarettes
CEMA: 2-Cyanoethyl mercapturic acid (MA of acrolonitrile)
CI: 95%-Confidence interva
CNO: Cotinine-N-1-oxide
CODS: Clinical oral dryness score
COex: Carbon monoxide in exhaled breath
COHb: Carboxyhemoglobin
Cot: Cotinine
Cot-gluc: cotinine-N-glucuronide
CRP: C-reactive protein (inflammation marker)
CSS: Cross-sectional study
CTX: C-terminal cross-links
D: Duration (of use of T/N products or NCPs)
DU: Persons who simultaneously use CCs and ECs (so called dual use)
EC: Electronic cigarettes
EN-RAGE: Extracellular newly identified RAGE binding protein
ENDS: Electronic nicotine delivery systems
FMUS: Full mouth ultasonic scaling
FS: Persons formerly using cigarettes (usually of CC, mostly termed former smoker or ex-smoker in the literature)
FU: Follow-up (in a prospective study
G: Group
GCF: Gingival crevicular fluid
GCol: Gingival color
GI: Gingival index
GM-CSF: Granulocyte-macrophage colony-stimulating factor (pro-inflamm. marker)
GSH-Px: Glutathione peroxidase
HNSCC: Head and neck scquamous cell cancer
HPRT: Hypoxanthine phosphoribosyltransferase
HTP: Heated tobacco product
Hypybut: 4-OH-4-(3-pyridyl)-butanoic acid
IL-1ß: Interleukine-1ß (pro-inflammatory cytokine)
IL-2: Interleukine-2 (pro-inflammatory cytokine)
IL-4: Interleukine-4 (anti-inflammatory cytokine)
IL-6: Interleukine-6 (pro-inflammatory cytokine)
IL-8: Interleukine-8 (anti-inflammatory cytokine)
IL-9: Interleukine-9 (anti-inflammatory cytokine)
IL-10: Interleukine-10 (anti-inflammatory cytokine)
IL-12p70: Interleukine-12p70 (?-inflammatory cytokine)
IL-13: Interleukine-13 (anti-inflammatory cytokine)
IL-RA: Interleukine-RA (anti-inflammatory cytokine)
IL-15: Interleukine-15 (?-inflammatory cytokine)
IL-18: Interleukine-15 (?-inflammatory cytokine)
INF-γ: Interferon-gamma (pro-inflammatory cytokine)
IQR: Inter-quartile range (25th – 75th percentile)
LA-QPCR: Long-amphicon quantitative polymerase chain reaction
LC-MS/MS: Liquid chromatography with tandem mass spectrometry
LDH: Lactate dehydrogenase (elevation can indicate oxidative stress, cell damage and cell death)
LRG1: Leucine-rich alpha-2-glycoprotein (BM for tumor vascularization)
LS: Longiturinal study
m: Month
MA: Mercapturic acid
MBL: Marginal bone loss
mi-RNA: micro-RNA (not coding RNA, but involved in gene regulation)
MN: Micronulei
MPO: Myeloperoxidase
MT: Missing teeth
N: Nicotine
NCot: Norcotinine
Nequ: Nicotine equivalents
NCP: Non-combustible (tobacco/nicotine) product (in this review: EC, HTP, ONP)
NHANES: National Health and Nutrition Examination Survey (USA)
N(ic): Nicotine
Nic-gluc: Nicotine-N’-glucuronide
Nic + 10: Nicotine and its 10 major metabolites Cot, 3-OH-Cot, Nic-gluc, Cot-gluc, 3-OH-Cot-gluc, NNic, NCot, NNO, CNO, Hypybut
NN: Nornicotine
NNN: N-Nitrosonornicotine
NNO: Nicotine-N-1’-oxide
NRT: Nicotine replacement therapy
ns: Not (statistically) significant
NS: Persons not using cigarettes (mostly termed ‘non-smokers’ in the literature)
nts: Nucleotides (in DNA)
NU: Persons not using any tobacco/nicotine products (also not in the past). Persons termed ‘never- smokers’ in the original study were classified as NU in this review)
NV: Person who is not vaping
OML: Oral mucosa lesions
ONP: Oral nicotine pouch
OPG: Osteoprotegerin
OR: Odd ratio
OT: Oral tobacco (and user)
P: Plasma
PATH: Population Assessment of Tobacco and Health (study in USA)
PBI: Papillary bleeding index (indicator of gingival inflammation)
PCot: Cotinine in plasma
PD: Probing depth
PG: 1,2-Propylene glycoöl
PGE2: Prostaglandin E2 (involved in inflammatory processes)
PI: Plaque index
PIBL: Peri-implant bone loss
PISF: Peri-implant sulcular fluid
POLB: (DNA) Polymerase-beta
POR: Prevalence odd ratio
PS: Plaque score
PY: Packyears
Q: Questionnaire
RAGE: Receptor for advanced glycation end products
RANKL: Receptor activator of NF-kappa B ligand
RBL: Radiographic bone loss
S: Saliva
SD: Standard deviation of the mean
SEM: Standard error of the mean
SGP: Subgingival plaque
SLT: Smokeless tobacco
SRP: Sealing and root paving
TIMP-1: Tissue inhibitor metalloproteinase-1
TNF-α: Tumornekrosefaktor-α (pro-inflammatory cytokine)
TNH: History of using tobacco/nicotine products
T/N: Tobacco/Nicotine (products, history, etc.)
U: Urine
WP: Waterpipe
w/o: With or without
