# Supplementary material for: Oral health risks in adults who use electronic nicotine delivery systems and oral nicotine pouches: a critical review of the literature and qualitative synthesis of the available evidence
Source: Harm Reduct J. 2024 Dec 31;21:229. doi: 10.1186/s12954-024-01147-y (PMC11687081; doi:10.1186/s12954-024-01147-y)
Supplement: Supplementary file 1 — Supplementary Material 1 [file 12954_2024_1147_MOESM1_ESM.docx]

Supplemental File to:

**“Oral health risks in adults who use electronic nicotine delivery systems and oral nicotine pouches: A critical review of the literature and qualitative synthesis of the available evidence”**

Gerhard Scherer, Nikola Pluym and Max Scherer

ABF Analytisch-Biologisches Forschungslabor GmbH, Semmelweisstr. 5, 82152 Planegg, Germany

Harm Reduction Journal

**Table S1: Selected studies (N = 52) for the review on oral effects in users of NCPs** (abbreviations see Section ‘Abbreviations`in the main document)

*(ordered according to internal ID #)*

| **ID #^1^** | | **Author, year (reference)** | **NCP (s) studied; pos./neg. control (+/-)** | **Study type and design; groups (G), numbers (N) and characteristics of subjects (gender, age)** | **History of tobacco/nicotine (T/N) products use (assessment methods and results)^2^** | **Study outcome and endpoints** | **Comments (limitations, weaknesses)** |
| --- | --- | --- | --- | --- | --- | --- | --- |
| 4 | | Akinkugbe et al., 2018 (1) | EC  +: CC  -: never users (NU) of EC or CC | Cross-sectional study (CSS); PATH (USA), 2013  13650 Adolescents (M/F)  G1: Current CC (3.2 %)  G2: Current EC (1.7 %)  G3: Current dual, CC/EC (1.4 %)  G4: Ever dual, CC/EC (7.1 %) | Q, self-reports  Prevalences: see cell on the left  Duration (D) of CC, EC or dual use not provided | -Dental problems (diagnosed by dentists in past year)  Prevalence odd ratios (POR) (CI) compared to NU:  -G1: 1.50 (1.18-1.90)  -G2: 1.11 (0.79-1.55)  -G3: 1.72 (1.24-2.38)  -G4: 1.43 (1.22-1.67) | -CSS (no causality)  -Only short NCP duration (adolescents)  -Only self-reports for NCP use |
| 5 | | Akram et al., 2021 (2) | EC  +: CC  -: none | Longitudinal study (LS):  Follow-ups (FUs): baseline (BL), 3 m, 6 m  -G1: EC (30)  -G2: CC (30) | Tobacco/NCP history: questionnaire (Q)  Duration of EC use (G1): ≥ 2 y  Long-term dose for EC: Session-years  Duration of CC use (G2): ≥ 2 y  Long-term dose for CC: Pack-years  NCP only use: self-report, not verified | Dental inspection of healthy and periodontitis sites in each subject: CAL, PD, MBL, PS  BMs in GCF: MMP-8, CTX;  At BL: CAL, PD, MBL, PS: EC ≈ CC  BL to FU changes: PD, CAL, MBL, CC: showed sign. higher periodontal worsening than EC group; PS: EC ≈ CC  CC and EC group showed sign. correlation between long-term dose and MMP-8, CTX, PD and CAL | -EC compliance not checked  -Small group sizes  -No negative (-) control |
| 7 | | Al-Aali et al., 2018 (3) | EC  +: none  -: NS (never smokers) | CSS, 92 Males having received implants  -G1: 47 habitual EC users, 35.8±6.2 y  -G2: 45 NS, 42.6±2.7 y | NCP/T/N History: Q, self-reports  -Duration of EC: ≥ 1 y (4.4±1.8y)  -6.5 EC sessions/d  -37.7 min/session  -CC, WP, SLT excluded  -Dual use not mentioned | Peri-implant parameters: PI, BOP, PIBL,  In prei-implant sulcular fluid: TNF-α, IL-1ß  -BOP: G2>G1(p<0.01)  -PD: G1>G2 (p<0.05)  -TNF-α: G1>G2 (p<0.01)  -IL-1ß: G1>G2 (p<0.01)  -PIBL: G1>G2 (p<0.05)  -PI: G1≈G2 (ns) | -CSS (no causality)  -Possible dual use not considered  -EC only use not verified  -No post CC use provided  -No dose-response provided (would be possible)  -LS urgently required (authors) |
| 8 | | Al-Hamoudi et al., 2020 (4) | EC  +: none  -: NS (never smokers) | LS: with BL and FU at 3 months  Subjects with moderate chronic periodontitis (under SRP treatment)  -G1: 36 EC, 47.7±5.8 y, M/F: 32/4  -G2: 35 NS, 46.5±3.4 y, M/F: 30/5 | NCP/T H: Q, self-reports  -Duration of EC: ≥ 1 y (3.3±0.5y)  -17.6±3.1 sessions/d  -8.4±1.5 puffs/session  -CC, WP, SLT excluded  -Dual use not mentioned  -All EC users were former smokers (CC), quitted 3 y prior and had 11.2±0.8 pack-y | Anti-inflamm BMs in GCF: IL-4, IL-9, IL-10, IL-13  At BL:  PI, PD, MBL, CAL, IL-4, IL-9, IL-10, IL-13 not different (ns) between G1 and G2  At 3 months (most probably treatment effects, not included in meta-analysis):  -G1: PI, GI, PD, CAL, MBL not different (ns) from BL  -G2: PI, GI, PD sign. reduced compared to BL  -G1 and G2: IL-4, IL-9, IL-10, IL-13 sign. increased, increase sign. higher in G2 than G1 | -CSS (no causality)  -Dual use likely (authors), but not reported  -No positive control  -No verification of EC only use |
| 9 | | Alazmi et al., 2021 (5) | EC  +: none  -: NS (never smokers) | CCS (8 years after receiving implants)  127 Subjects, M/F: 92/35  -G1: 63 EC (46/17), 34.3±1.2y  -G2: 64 NS (46/18), 35.1±0.5y | NCP/Tobacco history: Q, self-reports  -Duration of EC: 9.2±0.8y  -6.2±0.4 sessions/d  -3.5±0.2 puffs/session  -Dual use not mentioned  -No former tobacco habit provided | Peri-implant parameters:  PI, BOP, PD, CBL  -Parameters not different (ns) between G1 and G2 | -CCS (no causality)  -No positive control  -Only self-reports on the NCP/Tobacco history  -No verification of EC only use |
| 11 | | Alharthi et al., 2019 (6) | EC  +: CC  -: Never smokers (NS) | LS: Prospective study on FMUS patients with gingivitis: BL and FU at 3 and 6 months; 89 males  -G1: 30 Current CC, 36.4±2.8y  -G2: 28 EC, 32.5±4.8y  -G3: 31 NS, 32.6±3.5y | NCP/Tobacco history: Q, self-reports  -Duration of EC: 3.1±0.4y  --12.5±0.8 sessions/d  -Duration of CC: 10.4±1.8y  --9.3±4.6 cig/d  -Dual users and users of other T/N products were excluded | Clinical parameters: PI, BOP, CAL, PD  -Gingival inflammation following FMUS is sign. worse in G1 (CC) compared to G2 (EC) and G3 (NS)  -G2 ≈ G3 (ns), except BOP at BL  BL to FU changes: probably treatment effects (?) | -Only self-reports for the NCP/T history  -EC only use not verified  -Only males |
| 12 | Ali et al., 2022 (7) | | EC  +: CC  -: Never smokers (NS) | CSS, case-control study  -G1: Current CC (19), M/F=15/4, 52.6±6.1y  -G2: EC (18), M/F=12/6, 49.5±2.3y  -G3: NS (19), M/F=13/6, 50.7±2.2y, with periodontitis  -G4: NS (19), M/F=14/5, 48.1±1.3y, without periodontitis | NCP/T History: Q, self-reports  -EC user definition: at least once in last 30d  --Duration of EC: 12.5±0.8y  --25.1±3.5 sessions/d  --6.6±0.7 puffs/session  -Dual users excluded | Clinical markers for periodontal status: PI, CAL, PD, MBL, MT  Inflammation BM in saliva: IL-15, IL-18  Results:  -PI, CAL, PD, MT: higher in G1 (p<0.001), G2 (p<0.001), G3 (p<0.001) compared to G4  -PI, CAL, PD, MT, MBL: similar in G1, 2, 3  -IL-15, IL-18: higher in G1 and G2 (p<0.001) compared to G3 and G4; G3 > G3 (p<0.001) | -CSS (causality)  -Only self-reports on EC use  -EC only use not verified  -Small group sizes  -Dose-response not investigated (would be possible) |
| 13 | | Alizadehgharib et al., 2022 (8) | NP (3 or 6 mg N)  +: none  -: none | LS: Intervention study over 6 weeks, 60 snus users were asked to replace snus with ONP, visits at BL, 2, 4, 6 weeks  -M/F=39/21  -age: 31±10y | NCP/T History: phone interview, self-report, ONP were freely provided at each visit | Changes in oral mucosa lesions at the site of snus/ONP use  -Score and severity of lesions significantly decreased from BL to 6 weeks FU | -Compliance for ONP use not verified  -Only short ONP use investigated (6 weeks)  -No inclusion of a pos. (snus) or neg. (cessation) control |
| 14 | AlJasser et al., 2021 (9) | | EC  +: CC  -: NS (never smokers) | CSS with visits at BL, 1, 6, 12 months post treatment start (implants)  60 Patients, 18-70y:  -G1: 20 CC, M/F=12/8, 54.1y  -G2: 20 EC, M/F=9/11, 46.8y  -G3: 20 NS, M/F=10/10, 46.9y | NCP/T History: assessment of data not reported  -CC: at least 1y (past year)  --9.2±0.6 cig/d  -EC: at least 1y (past year)  --6.5±0.9 sessions/d | Peri-implant clinical parameters: gingival color, BOP, PD, PI;  pro-inflamm. BMs in saliva: IL-1ß, IL-6, MMP-8, TNF-α, TIMP-1  Treatment outcome:  -Order for sign. least favorable outcome over time:  G2 > G1 > G3 | -No information on NCP/T use assessment  -Dual use not considered  -Exclusive EC (EC only) use not verified |
| 15 | Alqahtani et al., 2019 (10) | | EC  +: CC, WP  -: NS (never smokers) | CSS: 102 Males with implants  -G1: 35 CC: 35.3±1.2y  -G2: 33 WP: 34.1±1.2y  -G3: 34 EC: 33.5±0.7y  -G4: 35 NS: 32.2±0.6y | NCP/T History: Q, self-reports  -CC: at least 1 y and 1 cig/d  --D: 10.2±4.1y; 9.3±0.6 cig/d  -WP: at least 1 y and 1 WP/d  --D: 8.3.2±0.4y; 5.1±0.3 WP/d  -EC: at least 1 y and 1 session/d  --D: 3.5±0.6y; 14.3±1.2 session/d  -Dual users were excluded  -EC and WP were all former CC users | Peri-implant clinical parameters: PI, PD, BOP; in PISF: cotinine  Outcome: G1/G2/G3/G4  **-**PI (%): 38.6^a,b^ /35.5^c,d^/27.2^a,c,e^ /12.6^b,c,d,e^  -BOP (%): 6.8^a^/7.9^b^/6.6^c^/19.8^a,b,c^  -PD (mm): 4.8^a,b^/4.4^c,d^/3.2^a,c,e^/0.8^b,d,e^  -Cotinine (ng/mL): 307^a^/276^b^/242^c^/2.2^a,b,c^  ^a,b,c,…^:same letters mean significant difference between groups (p<0.05) | -CSS (no causality)  -Only self-reports for NCP habit  -EC only use not verified |
| 16 | Alqahtani et al., 2018 (11) | | EC  +: CC, WP  -: NS (never smokers) | CSS: 160 Males, ≥ 30y old, with implants (≥3y ago),  -G1: 40 CC: 45.8±6.8y  -G2: 40 WP: 43.5±4.9y  -G3: 40 EC: 35.6±7.1y  -G4: 40 NS: 43.6±2.7y | NCP/T History: Q, self-reports  CC, WP and EC inclusion criteria: at least 10 times/d for >5y  -CC, D: 21.3±5.2y; 14.6±3.8 cig/d; 8.1±0.7 min/cig  -WP, D: 19.5±2.0y; 5.9±1.1 WP/d; 31.6±2.2 min/WP  -EC, D: 8.7±3.8y; 6.5±0.9 sess/d; 37.7±11.3 min/sess | Peri-implant clinical parameters: PI, PD, BOP, RBL; in PISF: TNF-α, IL-6, IL-1ß  Outcome: G1/G2/G3/G4  -PI (%): 67.4^a^/62.8^b^ /51.9^c^/34.1^a,b,c^  -BOP (%): 16.7^a^/18.4^b^/23.3^c^/38.9^a,b,c^  -PD (mm): 7.8^a,b^/7.0^c^/5.3^a,c,d^/4.4^b,c,d^  - TNF-α, IL-6, IL-1ß: G1^a^≈G2^b^>G3^c^>>G4^a,b,c^  (with G1, G2, G3 sign diff. from G4)  ^a,b,c,…^: same letters mean sign. difference between groups (p<0.01) | -CSS  -Only self-reports for NCP use  -Dual use not even considered  -EC only use not verified  -No former use of CC considered  -LS regarded as necessary (authors) |
| 17 | ArRejaie et al., 2019 (12) | | EC  +: CC  -: NS (never smokers) | CSS: 95 Males with implants  -G1: 32 CC: 40.4±3.5y  -G2: 31 EC: 35.8±6.2y  -G3: 32 NS: 42.6±2.7y | NCP/T History: Q, self-reports  -CC: at least 1 y (past year)  --D: 13.7±7.2y; 11.3 cig/d; 10.4 min/cig  -EC: at least 1 y (past year)  --D: 4.4±1.8y; 6.5 sessions/d; 37.7 min/session  -Dual users excluded | Peri-implant clinical parameters: PI, PD, BOP, MBL; in PISF: MMP-9, IL-1ß  Outcome: G1/G2/G3  -PI (%): 56.4^a,b^/43.5^a,c^/29.7^b,c^  -BOP (%): 18.4^a^/14.7^b^/39.8^a,b^  -PD (mm): 23.8^a^/15.9^b^/4.5^a,b^  -MBL (mm): 3.1^a,b^/1.2^a,c^/0.8^b,c^  -MMP-9 (ng/mL): 113^a^/88^b^/28^a,b^  -IL-1ß (pg/mL): 247^a^ /196^b^/378^a,b^  ^a,b,c,…^: same letters mean sign. difference between groups (p<0.01) | -CSS  -Only self-reports for NCP use  -EC only use not verified  -No former use of CC considered  -LS regarded as necessary (authors) |
| 18 | | Atuegwu et al., 2019 (13) | EC  +: None  -: NU (never EC users) | LS: PATH, 2013-2016 (waves 1, 2, 3)  Subjects reporting no ‘gum disease’ at BL (wave 1), 18-55+ y old:  -G1: 9632 NU at all 3 waves, 44.4 % M  -G2: 329 longitudinal EC (all 3 waves), 53.2 % M  -G3: 8298 non-longitudinal EC: 52.3 % M | T/N History: Q, self-reports  Longitudinal CC (%)/FS (%)  -NU: 4.3/20.5  -Longitudinal EC: 38.6/38.6  -Non-longitudinal EC (no daily use): 40.1/14.5 | Self-reported oral health problems:  Odd ratio (CI) compared to G1 (NU):  -New gum disease: G2: 1.76 (1.12-2.76)  G3: 1.09 (0.87-1.35)  -BLT: G2: 1.67 (1.06-2.63)  G3: 1.10 (0.91-1.33)  -Any periodontal. disease:  G2: 1.58 (1.06-2.34)  G3: 1.09 (0.93-1.29)  No effects over time (BL to FU) reported | -Dual use for G2 not considered, let alone verified  -Only self-reports (T/N history and diagnosis), recall bias  -No positive control |
| 19 | | Bardellini et al. 2018 (14) | EC  +: None  -: Former smokers (FS) | CSS: Prospective case-control, 90 patients with OML (according to WHO), ≥18 y  -G1: 45 FS, M/F=22/23, 47±11y  -G2: 45 EC, M/F=41/4, 47±10y | T/N History: Q (?), self-reports (?)  -FS: quit CC 0.5-2 y prior to BL  -EC: at least 6 months use | OML (11 lesions)  -Total OML: No sign. difference between G1 and G2  -3 single OML (nicotine stomatitis, hairy tongue, hyperplastic candidiasis): sign. higher in G2 (EC) compared to G1 (FS) | - Unclear assessment of T/N history  -Dual use in G2 not considered or verified  -No positive control  -Small group sizes |
| 20 | BinShabaib et al, 2019 (15) | | EC  +: CC  -: NS (never smokers) | CSS: Healthy subjects  -G1: 46 CC, M/F=43/3, 44.3±3.5y  -G2: 44 EC, M/F=42/2, 36.5±1.7y  -G3: 45 NS, M/F=41/4, 40.6±3.3y | T/N History: Q, self-reports  -CC: ≥5 cig/d, D: ≥1y, 14.2±0.6 PY, 5.2±0.6 min/cig  -EC: ≥1 times/d, D: 9.4±2.6y, 20.3±3.5 min/session  -NS: never any tobacco product  -Dual users excluded | Clinical periodontal status: PI, BOP, PD, AL, MBL  Cytokines in GCF: IL-1ß, IL-6, TNF-α, MMP-8, IFN-γ  Outcome G1 / G2 / G3:  -PI, PD, AL: G^a^ > G2 > G3  -BOP: G1 < G2 < G3^a^  -MBL: G^b^ > G2 > G3  -MT: G1 ≈ G2 ≈ G3  - IL-1ß, IL-6, TNF-α, MMP-8, IFN-γ:  G^a^ > G2 > G3  ^a^: diff. from other two groups (p<0.05)  ^b^: diff. from other two groups (p<0.01) | -CSS  -Only self-reports  -Small group sizes  -Assessment of dual use in G2 unclear |
| 21 | | Cheng et al., 2022 (16) | EC  +: CC  -: NS | LS: 6 month FU with monthly visits  Healthy subjects, ≥18 y  -G1: 20 EC, M/F/?=8/11/1, 18-62y  -G2: 20 NS, M/F=6/14, 21-55y  -G3: 8 CC, M/F=6/2, 28-59y | T/N History: Interview/Q?, habit verified with BMs: CEMA, Nequ, COex  -EC: D: ≥3m, 4d/week, Nequ > 3 nmol/mL, CEMA<27 pmol/mL  -NS: <6 ppm COex  -CC: >8 ppm COex, CEMA>27 pmol/mL | Acrolein-DNA adduct in brushed oral cells at 3 visits:  Adduct levels pmol/µmol Guo, median (range):  -G1: 179 (5-793)^a ,b^  -G2: 21 (5-539)^a,c^  -G3: 446 (158-5830)^b,c^  Adducts sign. correlate with age in G1 and G2  BL vs FU: not evaluated | -Strength: compliance verified with suitable BMs  -Small group sizes |
| 22 | Cho, 2017 (17) | | EC  +: CC  -: NU (never EC users) | CSS: 65528 Adolescents (Web survey in Korea), 14.99 ±1.74y, 51.6 % males  -G1: 297 EC (daily)  -G2: 1259 EC (1-29 d in past month)  -G3: 3848 former EC  -G4: 60124 NU | T/N History: Q, self-repots  -G1: contains 187 CC users  -G2: contains 486 CC users  -G3: contains 839 CC users  -G4: contains 411 CC users | Self-reported oral symptoms, adjusted OR (CI) vs G4:  -Cracked or broken teeth:  --G1: 1.65 (1.19-2.27)  --G2: 1.26 (106-1.51)  --G3: 1.16 (1.05-1.30)  -Tongue and/or inside cheek pain:  --G1: 1.54 (1.05-2.26)  -Gingival pain and/or bleeding:  --No association with EC use  Nicotine-free EC users showed no consistent differences from EC with nicotine  Daily CC users showed lower (!), mostly ns OR compared to daily EC use | -CSS  -Dual users are included in G1-G3  -No separate exclusive EC (EC only use) group  -CC results indicate possible issue with CSS approach |
| 23 | | Chopyk et al., 2021 (18) | EC  +: None  -: NU (never EC or CC users, NSNV) | LS: 2 weeks reduced use of EC  -G1: 12 NU, 21y, M/F=3/9  -G2: 12 EC, 21y, M/F=12/0 | T/N History: Q, self-reports  -EC (mean (CI)):  --D: 1.5 (0.9-2.0)y  --6.7 (5.7-7.0) d/week  --N in e-liquid: 21.3 (5.1-37.6) mg/mL  --5.4 (0.6-12.2) mL/d (e-liquid consumption) | Oral microbiome (in saliva, buccal and nasal swabs):  -Sign. shift in G2 to pathological changes  -Reduction in EC use: sign decrease in changes in salivary microbiome of G2 (not in buccal samples) | -Small group sizes  -Many influencing factors on microbiome (diet, alcohol, oral hygiene)  -No use of other T/N products assessed  -No positive control  -Strength: dose-response shown |
| 24 | Stewart et al., 2018 (19) | | EC  +: CC  -: NS | CSS: 30 Subjects, age (range)  -G1: 10 EC, 29 (21-37)y, 9 Males  -G2: 10 CC, 35 (30-45)y, 10 Males  -G3: 10 NS, 31 (28-36)y, 9 Males | T/N History: Q, self-reports  -EC:  --N-content: 9 (6-12) mg/mL  --D: 3 (2-4) y (median)  --COex, median (IQR): 3 (3-4) ppm  -CC:  --14 (10-19) cig/d  --COex,: 14 (10-19) ppm  -NS, COex,: 1 (1-2) ppm | Oral and gut microbiome (fecal, buccal swab and saliva samples):  -CC (but not EC) is sign. associated with shifts in bacterial profiles in studied samples | -CSS  -Small groups  -COex indicates possible CC use in G1  -Authors consider bias in self-reported T/N use |
| 25 | Cichonska et al., 2019 (20) | | EC  +: CC  -: NS | CSS: 120 Healthy subjects, 20-30y  -G1: 40 EC  -G2: 40 CC  -G3: 40 NS | T/N History: Q (?), sel-report  -EC: D: ≥6 months, ≥50 times/d (puffs?)  -CC: D: ≥6 months, ≥10 cig/d  -Dual users excluded | Anti-bacterial properties of saliva:  G1 / G2 / G3, µg/mL, medians:  -IgA: 1.7^a^ / 1.8^b^ / 6.5^a,b^  -Lysozyme: 1.6^a^ / 1.4^b^ / 4.8^a,b^  -Lactoferrin: 7.1^a^ / 1.4^a,b^ / 5.6^b^  ^a,b^: same letter means significant difference between groups (p<0.05) | -CSS  -No verification of EC only use  -Gender not specified  -Longer and larger studies required (authors) |
| 26 | Cichonska et al., 2022 (21) | | EC  +: CC  -: NS | CSS: 128 Healthy subjects, 20-30y  -G1: 40 EC  -G2: 39 CC  -G3: 49 NS | T/N History: Q (?), self-reports  -EC: D: ≥6 months, multiple times/d  -CC: D: ≥6 months, ≥10 cig/d  -Dual users excluded | Physicochemical properties of saliva: pH, total proteins (?), calcium (mM), phosphates (mM)  G1 / G2 / G3, medians same letter (^a^): p<0.05  -pH: 7.0 / 7.0 / 7.5  -Total proteins: 1.8 / 2.1^a^ / 1.7^a^  -Calcium: 8.8^a^ / 0.6 / 0.6^a^  -Phosphates: 4.1 / 3.0^a^ / 2.9^a^ | -CSS  -No verification of EC only status  -No gender provided  -Authors have problems with units  -Larger and longer studies required (authors) |
| 28 | Faridoun et al., 2021 (22) | | EC  +: CC  -: NS | CSS: 64 Subjects, M/F=37/27, 55.7±16.8 (28-83)y, pilot study  -G1: 15 NS  -G2: 18 CC  -G3: 15 EC  -G4: 16 Dual (mixed) | T/N History: Assessment not provided | Biomarker profiles in saliva:  Pro-inflammation cytokines: IL-6, IL-8, IL-1ß, TNF-α  Anti-inflammation cytokines: IL-10, IL-1RA, CRP  ANOVA:  -Anti-inflammation BMs: No sign. diff. between groups  -Pro-inflammation BMs: Sign. elevation in G3 for IL-1ß and TNF-α | -CSS  -No information on T/N History  -Small group sizes |
| 29 | Franco et al., 2016 (23) | | EC  +: CC  -: NS | CSS: 65 Subjects, > 18y  -G1: 23 CC, M/F=10/13, 47.6 (23-73)y  -G2: 22 EC , M/F=12/10, 57.7 (27-73)y  -G3: 20 NS, M/F=11/9, 46.7 (23-74)y | T/N History: Q, self-reports  -EC: D: ≥ 6 months, no CC in last 6 months  -CC: D: ≥ 6 months | MN in scraped oral mucosa cells  Total MN per 1000 cells, mean±SD:  -G1 (CC): 0.088±0.0058  -G2 (EC): 0.028±0.024  -G3 (NS): 0.012±0.0056  No sign. differences between groups | -CSS (authors state ‘prospective study’?)  -No verification of EC only use  -Authors conclude that EC use is safe with respect to oral cancer risk |
| 30 | Ganesan et al., 2020 (24) | | EC  +: CC  -: NS (never smokers) | CCS: 127 Periodontally healthy subjects, 21-35y, gender and age was matched for groups  -G1: 25 CC  -G2: 25 NS  -G3: 20 EC  -G4: 25 EC, former CC (FS)  -G5: 28 Dual users | T/N History: Q, self-reports  -CC: ≥5 PY, no ECs  -NS: <100 cig in lifetime, 0 cigs in past year  -EC: daily use in ≥3 past months, D: 7 months (mean), ≥1 cartridge/d or mL/d  -FS: quit ≥1y ago | Oral microbiome, cytokines in GCF: INF-γ, IL-2, IL-4, IL-6, IL-8, IL-10, GM-CSF, TNF-α  Outcome:  -IL-2, IL-6, GM-CSF, TNF- α, INF- γ: sign. higher in EC groups vs NS  -IL-10: sign. lower in EC groups vs NS  -Similar cytokine result in CC (but other mechanism)  -Pathogen over-representation and higher virulence in EC groups  -PG and G are important catalysts in transformation of biofilm architecture within 24 h EC use | -CSS  -Different mechanism for microbiome changes postulated for EC and CC  -No long-term effects of ECs assessed |
| 32 | | Holliday et al., 2019 (25) | EC  +/-: CC (switched to EC.)  -: NS (quit) | LS: Pilot study over 22 months, 80 patients smoking CC with periodontitis, 58 completed  -G1: 40 EC (intervention group), M/F=18/22, 44.0±11.8y  -G2: 40 stopped (control), M/F=20/20, 44.6±9.5y | T/N History: Q, self-reports  -G1 at BL/6 m (FU):  --COex: 23.0/12 ppm  -G2 at BL/6 m (FU):  --COex: 18.1/6 ppm | Oral health parameters: PD, BOP, clinical oral dryness score (CODS):  BL to FU: PD: similar improvement after 6 months (FU) compared to BL in G1 and G2; CODS: no change in EC, decrease in NU    (No final evaluation of later time-points) | -No final results reported  -There are probably CC users in both groups (according to COex) |
| 33 | Huilgol et al., 2019 (26) | | EC  +: CC  -: No CC or no EC | CSS: Behavioral Risk Factor Surveillance System (BRFSS), 456343 adults, 43.4% males, 18-65+ y:  -G1: 4957 EC daily  -G2: 10052 EC intermittent  -G3: 67003 CC  -G4: 387000 NS (no CC)  -G5: 441324 no EC | T/N History: Q, self-reports  -EC: daily or intermittent use within last 30 d  -CC: current use within last 30 d | Self-reported poor (loss of teeth, periodontal disease) or good oral health  Outcome: OR for poor vs good oral health (CI):  -G1 vs G5: 1.78 (1.39-2.30)  -G2 vs G5: 1.08 (0.87-2.32)  -G3 vs G4: OR (CI) similar to G1 vs G5  -Strong increase of OR with age | -CSS  -Only self-reports for T/N History and oral symptoms  -EC only use not verified  -Dual use not considered  -Probably very short EC use |
| 34 | Ibraheem et al., 2020 (27) | | EC  +: CC, WP  -: NS | CSS, case-control study with 120 male subjects  -G1: 30 CC, 46.5±5.3y  -G2: 30 WP, 45.5±4.4y  -G3: 30 EC, 45.6±3.6y  -G4: 30 NS, 43.8±1.7y | T/N History: Q, self-reports  -CC: D=18.3±2.8y, 12.6±1.5 cig/d, 8.3 min/cig  -WP: D=15.6±2.2y, 5.5±0.7 WP/d, 22.6±5.5 WP/d  -EC: D=6.4±2.2y±, 15.4±0.3 sessions/d, 20.5 min/session | Clinical periodontal parameter: PI, BOP, PD, CAL, MBL  In GCF: RANKL, OPG  Outcome:  -All clinical parameters: CC≈WP≈EC>>NS*  (reverse for BOP)  -RANKL, OPG: CC≈WP>EC>>NS*  *: sign. different from the other 3 groups | -CSS  -Dual use not considered  -Only self-reports  -No verification of EC only use |
| 35 | Irusa et al., 2022 (28) | | EC  +: None  -: NU (of EC) | CSS: 13098 Patients, 47.9 % males, 16-40+ y  -G1: 91 EC (EC yes)  -G2: 13007 NS (EC no) | T/N History: Q, self-reports  Only EC ‘yes’ or ‘no’ differentiation | Risk of caries:  Outcome: QR (CI), EC ‘yes’: 1.0 (ref)  -EC ‘no’: 0.36 (0.22-0.60)  -OR increased with age  -OR sign. higher in males | -CSS  -Only few (0.69% EC users)  -Social desirability bias (authors)  -Dual use not considered  -No verification of EC only use  -No CC group!?  -Longitudinal study necessary (authors) |
| 36 | Javed et al., 2017 (29) | | EC  +: CC  -: NS (never smokers) | CSS: 94 Males  -G1: 33 CC, 41.3±2.8y  -G2: 31 EC, 37.6±2.1y  -G3: 30 NS, 40.7±1.6y | T/N History: Q, self-reports  -EC: no FS, ≥12 months EC, D=2.2±0.3y, 6.8±0.8 times/d  -CC: ≥12 months, ≥5 cig/d, D=5.4±1.6y, 13.8±2.6 cig/d  -NS: never any tobacco use  Dual user were excluded | Self-reported oral symptoms: Gingival pain (GP),  Clinical parameters: PI, BOP, PD, CAL, MBL, MT  Outcome: CC / EC / NS  -MT: 5.1 / 3.8 / 3.3  -PI (%): 52.1^a,b^ / 23.3^b^ / 21.4^a^  -BOP (%): 5.8^a,b^ / 6.4^b^ / 27.5^a^  -PD (mm): 29.3^a,b^ / 5.1^b^ / 5.6.4^a^  -CAL (mm): 2.1 / 1.1 / 0.8  -MBL (mm): 2.6 / 2.0 / 2.1  -GP: Sign. more frequent in G1 than in G2 and G3  ^a,b^: same letter: sign. difference (p<0.05) | -CSS  -No verification of EC only use  -Only self-reports  -Only short-term EC use |
| 37 | Jeong et al., 2020 (30) | | EC  +: CC  -: NU (of CC or EC) | CSS: Korean NHANES, 2013-2015, 13551 Subjects, 19-60+ y, M+F  -G1: 222 EC, 35.8 % males  -G2: 2320 CC, 44.0 % males  -G3: 2667 FS, 41.9 % males  -G4: 8342 NU, 25.3 % males | T/N History: Q, self-reports  -EC, CC, FS, NU status only by ‘yes’ / ‘no’ answers  -No duration (D), use pattern or daily consumption provided | Periodontal status: diseased vs healthy  Outcome: adjusted OR (CI) vs NU  Males:  -G1: 2.34 (1.52-3.59)  -G2: 2.17 (1.76-2.68)  -G3: 1.28 (1.05-1.56)  Females:  -G1: 2.27 (0.89-5.80)  -G2: 1.73 (1.32-2.27)  -G3: 1.00 (0.74-1.34) | -CSS (cause, effect, directionality)  -Dual use not considered  -No verification of EC only use  -Recall and social desirability bias (authors)  -No details for T/N use pattern |
| 38 | Karaaslan et al., 2020 (31) | | EC  +: CC  -: FS (CC at least 12 months prior to study) | CSS: 57 Patients with periodontitis, 29.39y  -G1: 19 CC, M/F=12/7, 35.3±2.3y  -G2: 19 EC, M/F=13/6, 34.7±2.4y  -G3: 19 FS, M/F=11/8, 35.6±2.0y | T/N History: Q, self-reports  -FS: quit ≥12 months prior to study, ≥10 cid/d for ≥10y  -CC: ≥10 cid/d for ≥10y, D=14.0±3.0y, 13.7±3.7 cig/d  -EC: all were FS (≥10 cig/d for ≥10y), ≥12 months EC use  Dual and any other T/N users were excluded | Clinical periodontal parameters: CAL, PD, GI, PI  In GCF: volume, IL-8, TNF-α, GSH-Px, 8-OH-dG  Outcome: G1 / G2 / G3  -GI: 1.53^a^ / 1.81^a^ / 2.08^a^  (other clin. param. were not sign. diff. between groups)  GCF parameters:  -Volume (µL): 1.63^a^ / 1.82^a^ / 1.95^a^  -IL-8 (pg/µL): 70.5^a^ / 77.1^a^ / 80.1^a^  -TNF-α (pg/µL): 4.2^a^ / 3.4^a^ / 3.0^a^  -GSH-Px (U/µL): 6.4^a^ / 6.5^b^ / 6.7^a,b^  -8-OH-dG (ng/µL): 0.53 / 0.53 / 0.53  Same letter means sign. diff. (p<0.05 or better) | -CSS  -EC only use not verified  -Small sample sizes  -Only self-reports  -Short EC duration (~2y)  -Longitudinal studies required (authors) |
| 41 | Miluna et al., 2022 (32) | | EC, NP  +: Swedish snus, CC  -: NU (not any T/N product) | CSS: 76 Healthy subjects, M/F=38/38  -G1: 12 NP and snus, M/F=10/2, 25.1y  -G2: 19 CC, M/F=10/9, 24.7y  -G3: 8 EC, M/F=3/5, 23.0y  -G4: 37 NU, M/F=15/22, 24.5y | T/N History: Q, self-reports  T/N users: for at least 2 y | Saliva BMs: IL-6, IL-1, TNF-α, LRG1  Mucosa changes were documented  Outcome:  -Mucosa changes were primarily observed in snus users  -Pro-inflamm. BMs: G1>G3>G2>G4 (stat. Significance?)  -LRG1: not suitable (according to authors)  -IL-6 correlated best with mucosa changes | -CSS  -Only self-reports  -No clear separation of NCPs possible  -Small group sizes  -No stat. sign. reported |
| 42 | Mokeem et al., 2018 (33) | | EC  +: CC, WP  -: NU (never smokers or SLT users) | CSS: 154 Males  -G1: 39 CC, 42.4±5.6y  -G2: 40 WP, 44.7±4.5y  -G3: 37 EC, 28.3±3.5y  -G4: 38 NU, 40.6 ±4.4´5y | T/N History: Q, self-reports  -CC: ≥5 cig/d for ≥12 months, 4.8±0.3 min/cig, D=17.2±2.5y  -WP: ≥1 WP/d for ≥12 months, 4.3±0.5 WP/d, 17.5±3.4 min/WP, D=14.6±5.7y  -EC: ≥1 session/d for ≥12 months, 9.2±1.4 sessions/d, 8.1±1.3 min/session, D=3.1±0.4y  -NU: never CC, EP, SLT  Dual users excluded | Clinical parameters: PI, BOP, PD, CAL, MBL  In saliva: cotinine, IL-1ß, IL-6  Outcome: CC / WP / EC / NU:  -PI: G1^a^ ≈ G2^b^ > G3 ≈ G4^a,b^  -BOP: G1^a^ ≈ G2^b^ ≈ G3^c^ < G4^a,b,c^  -PD, CAL, MBL: G1^a^ ≈ G2^b^ > G3^a^ > G4^a,b^  -IL-ß, IL-6: G1^a,b^ ≈ G2^c,d^ >> G3^a,c^ ≈ G4^b,c,d^  (Same letter (^a,b,c^) indicates stat. sign. (p<0.05) | -CSS  -Only self-reports  -No verification of EC only use  -Strength: EC were not FS! |
| 43 | Park et al., 2023 (34) | | EC  +: None  -: NU | CSS: 150 Subjects, 18-34y  -G1: 75 EC, 23.8y, M/F=61/14  -G2: 75 NU, 24.4y, M/F=55/20 | T/N History: Q, self-reports  No tobacco use in last 3 months for G1 and G2  -EC: ≥6 months EC use and pos. cotinine urine test  -NU: No EC use last 90d and neg. cotinine urine test | Saliva and subgingival microbiome  Outcome: G1 *vs* G2:  -OR for gingival inflamm.: 2.47 (p<0.01)  -Sign increase of α-diversity and disparity in ß-diversity (microbial dysbiosis in the direction of periodontal disease) | -CSS  -No pos. control  -EC only use not verified  -Short duration of EC use to be assumed |
| 44 | Pushalkar et al., 2020 (35) | | EC  +: CC  -: NS (never smokers) | CSS: 119 Healthy subjects, ≥21y  -G1: 39 NS, M=56.4%  -G2: 40 Current EC, M=80.0%  -G3: 40 Current CC, M=77.5% | T/N History:  Saliva cotinine and COex for T/N status  -CC: ≥10 cig/d for ≥12 months, cot: 525 ng/mL, 11 cig/d (median), COex: 18.8±9.4 ppm  -NS: Never CC in lifetime, cot: 11.1 ng/mL COex: 1.8±2.3 ppm  -EC: never CC, 0.5-1 EC/d, COex: 5.1±6.9 ppm | Oral microbiome: ß-diversity in saliva  In saliva: IFN-γ, IL-1ß, IL-2, IL-4, IL-6, IL-8, IL-10, IL-12p70, IL-13, TNF-α  Outcome: NS / CC / EC:  -BOP (%): 53 / 65 / 57 (ns)  -PD (mm): 2.7^a^ / 3.3^b^ /3.0^a,b^  -INF- γ, IL-6: CC^a^ ≈ EC > NS^a^  -TNF- α: NS^a^ > EC^b^ > CC^a,b^  All other cytokines: ns  ^a,b^: same letter means sign. diff. between groups  -EC modulated oral microbiome and increased oral pathobionts (sign. diff. to CC and NS) | -CSS  -COex indicates some CC use in EC group  -Dual use not considered  -EC only use not verified |
| 45 | Reeve et al., 2023 (36) | | EC  +: None  -: NS | CSS: 47 Healthy subjects  -G1: 24 EC, M/F=13/11, 31 ±7y  -G2: 23 NS, M/F=12/11, 34 ±8y | T/N History: Q, self-reports  EC status check with nicotine/cotinine in urine  -EC: D=2±1y, 31±7 sessions/d  EC group included 7 dual users | Histology of epithelium, lamina propria and basal layer; RNA transcriptome (from buccal mucosa biopsies)  Outcome:  -Histology of G1 appeared normal  -mRNA and miRNA: no modifications compared to G2  -Dual used: No difference to EC only use | -CSS  -Other than epithelial cells included (dilution of epithel)  -Long-term study required (2y too short)  -No pos. control  -EC groups includes 29% dual users! |
| 46 | Schwarzmeier et al., 2021 (37) | | EC  +: CC  -: NS, FS | CSS: Healthy subjects, 17-80y  -G1: 20 EC, M/F=14/6, 41.5 ±13.0y  -G2: 22 CC, M/F=10/12, 51.5 ±11.3y  -G3: 22 FS, M/F=13/9, 58.9 ±10.2y  -G4: 27 NS, M/F=13/14, 56.5 ±12.9y | T/N History: Q, self-reports  -EC: ≥5 months of EC use, 23.3±15.5 ECs/d (puffs?), D=21.6±14.2y, COex=4.6±3.6ppm  -CC: 17.1±9.8 cig/d, D=33.1±11.9y, COex=10.1±8.7 ppm  -FS: 18.6±11.4 cig/d, D=39.9±13.2y, COex=1.7±0.8ppm, quit 1-2y before | MN and meta-nuclear anomalies in oral mucosa cells  Outcome:  -MN, Karyorrhexis: G2^a^ > G1 > G3 > G4^a^  -Karyolysis: G1^a,b^ > G2^c^ > G3^a^ > G4^b,c^  -Binucleation: G1^a,b^ >> G2 >> G3^a^ > G4^b^  -Broken egg: G1^a,b,c^ >> G2^a^ >> G3^b^ > G4^c^  -Nuclear bud: G1^a,b^ > G2 >> G3^a^ ≈ G4^b^  ^a,b,c^: same letter means sign. diff. between groups | -CSS  -Small group sizes  -Dual use not considered  -Unrealistic D for G1 (error?)  -Dual use is highly likely for G1  -Alcohol use is highest in G1 (strong upward bias) |
| 47 | | Tatullo et al., 2016 (38) | EC  +: None  -: None | LS (Pilot study): BL (0), FU: T1 (60 d), T2 (120 d):  110 EC users for ≥4 months, M/F=89/21, 31 ±9y  -G1: 60 EC with ≤10y CC  -G2: 50 EC with >10y CC | T/N History: Q, self-reports  -CC (prior to LS): 0.8-1 mg N/cig, ~20 cig/d  -EC: 0.25 mL e-liquid (?), 18 mg N (?), 4.5 mg N/cycle, 20 ECs/d  In 20% of subjects (N=22) COex at BL, T1, T2 | Clinical parameters: PI, PBI  Outcome: BL / T1 / T2  -PI: --G1: 0.9 / 0.8 / 0.0  --G2: 2.13 / 1.63 / 0.25  -PBI: --G1: 0.4 / 0.2 / 0.0  --G2: 1.25 / 0.25 / 0.0  -COex: Use of CC (dual use) decreased from BL to T1 to T2 | -Strength: Dual use was checked (at least partly)  -COex suggests some dual use  -Results show that former smoking is important for periodontal status  -No pos. and neg. controls |
| 48 | | Thomas et al., 2022 (39) | EC  +: CC  -: NU (never CC or EC) | LS: 84 Subjects with at least mild periodontitis, ≥21y, FU: BL (=0), T1 (6 months)  -G1: 27 CC, 81.5% males, ~50y  -G2: 28 EC, 78.6% males, ~38y  -G3: 29 NU, 55.2% males, ~35y | T/N History: Q, self-reports  COex and cotinine in urine determined in all subjects  -NU: never used CC or EC  -CC: ≥10 cig/d for ≥12 months  -EC: ≥0.5-1 ECs/d for ≥6 months, exclusion if COex > 7ppm | Periodontal condition: PD  Cytokines and microbiome in SGP  Outcome:  -FPD: G1^a^ > G2 ≈ G3^a^  -α-Diversity: similar increase from BL to T1 in all groups  -EC use promotes a unique periodontal microbiome (as a stable state between CC und NU), presenting an oral health challenge  -Cytokines (BL and FU samples for each cohort):  --IFN-γ, IL-2, IL-10: G2^a^ > G1 ≈ G3^a^  --IL-1ß: G1^a,b^ > G2^b^ ≈ G3^a^  --TNF-α: G2^a,b^ > G3^a^ > G1^b^  --IL-4: G1^a^ > G2^a^ ≈ G3  --IL-6, IL-8, IL-12p70, IL-13:  G1 ≈ G2 ≈ G3 | -Strength: EC status verified with COex  -COex suggests some dual use in EC group  -Small group sizes  -Cytokine results somewhat confusing |
| 50 | Tommasi et al., 2023 (40) | | EC  +: CC  -: NU | CSS:  -G1: 24 EC (never CC), M/F=20/4, 24.3±0.8y, D=2.9±0.4y  -G2: 24 CC, M/F=20/4, 26.0±0.7y  -G3: 24 NU, M/F=20/4, 25.3±0.6y | T/N History: Q, self-reports;  PCot, COex, COHb (calculated form COex)  -EC: never CC, D=2.9±0.4y, cumulatie e-liquid: 5780 mL/life-time, COex: 2.0±0.3 ppm, COHb: 0.9± 0.07%, PCot: 84.9±3.1 ng/mL  -CC: D=7.3±1.1y, PY=3.1±0.6, COex: 12.0±1.6 ppm, COHb: 2.5±0.2%, PCot: 76.7±8.6 ng/mL  -NU: COex: 1.9± 0.3 ppm, COHb: 0.9± 0.05%, PCot: 2.6±0.1 ng/mL | Oral epithelial cells: DNA damage in POLB and HPRT genes (analyzed with LA-QPCR)  -POLB: G1^a^ ≈ G2^b^ > G3^a.b^  -HPRT: G1^a^ ≈ G2^b^ > G3^a.b^  ^a,b^: same letter means sign. diff. between groups  Dose-response observed for G1 and G2 | -CSS  -Small group sizes  -Strength: Verification of self-reports for T/N use by BOEs |
| 51 | Tommasi et al., 2019 (41) | | EC  +: CC  -: NS | CSS:  -G1: 42 EC, M/F=34/8, 28±1.3y,  -G2: 24 CC, M/F=19/5, 42±2.8y  -G3: 27 NS, M/F=16/11, 24±1.7y | T/N History: Q, self-reports; PCot, COex, COHb (calculated form COex) for verification  -EC: cumulative e-liquid: 5370 mL/life-time, PCot: 115.0 ±8.5 ng/mL  -CC: PY=12.3 ±2.5, PCot: 122.0 ±10.8 ng/mL  -NS: PCot: 2.5 ±0.1 ng/mL | Oral epithelial cells: Gene dysregulation  Outcome:  -G1 and G2 showed sign. aberrant transcripts from G3  -G2 had more differently expressed transcripts than G1  -Functional network analysis: G1 and G2 dysregulated genes point in the direction of cancer | -CSS  -Elevated COex (COHb) in G1 indicate some use of CC  -Probably short duration of EC use (1.5-3y ?) |
| 52 | Vemulapalli et al., 2021 (42) | | EC  +: None  -: None  (at least no evaluation of pos./neg. controls) | CSS: NHANES 2017-2018, 4618 subjects evaluated, ≥18y, 48.1% males  -G1: 247 Current EC  -G2: 700 Former EC  -G3: 3671 Never EC  -G4: 803 Current CC  -G5: 1089 Former CC (FS)  -G6: 2726 NS  -G7: 120 Current Dual  -G8: 561 Former Dual  -G9: 3937 Never Dual | T/N History: self-reports (at home interviews)  -EC:  --Current: ever and in last 30d  --Former: ever and not in last 30d  --Never: not ever  -Dual:  --Current: current EC + current CC  --Former: current or former EC and FS; current CC or FS and former EC  -- Never: any CC and never EC; any EC and never CC | Untreated caries (coronal caries, N=928, 17.3 %)  Adjusted OR (CI):  -Current EC vs never EC: 1.69 (1.24-2.29)  -Former EC vs never EC: 1.47 (1.16-1.87)  -Current Dual vs never Dual: 2.43 (1.36-4.36)  -Former Dual vs never Dual: 1.57 (1.14-2.15) | -CSS (with all its problems)  -Highly likely that current EC is not exclusive EC (EC only)  -No OR for CC groups (no pos. control)  -Reference groups are no NU (neither EC nor CC)  -Prospective studies needed (authors) |
| 53 | Verma et al., 2021 (43) | | EC  +: CC  -: NS | CSS: 60 Adults, 18-80y, M/F=38/22  -G1: 14 NS (never CC)  -G2: 16 Current CC  -G3: 14 Dual  -G4: 16 EC | T/N History: Q, self-reports | Stimulated saliva:  Pro-inflammatory BMs: IL-1ß, IL-6, IL-8, TNF-α, CRP; anti-inflammatory BMs: IL-10, IL-1RA  Clinical parameters: PI, GI  Outcome:  -CRP: G1 < G2 ≈ G3 ≈ G4 (ns)  -IL-10, IL-1RA: G1 ≈ G2 ≈ G3 ≈ G4 (ns)  -TNF-α: G1 < G2 ≈ G3 ≈ G4 (p<0.001)  -IL-1ß: G1 ≈ G2 ≈ G3 < G4 (p<0.05)  -IL-6, IL-8: G1 ≈ G2 ≈ G3 ≈ G4 (ns) | -CSS  -Small group sizes  -Duration of use not considered (authors)  -LS needed (authors) |
| 54 | Vohra et al., 2020 (44) | | EC  +: CC  -: NS (never smokers) | CSS: 105 Healthy males  -G1: 28 CC, 33.3±2.2y  -G2: 26 EC, 31.6±2.4y  -G3: 25 JuuL, 32.1±1.7y  -G1: 26 NS, 33.5±1.4y | T/N History: Q, self-reports  -CC: ≤1 pack/d or ≤20 cig/d, 6.1±0.5 PY  -EC: ≥1 times/d, D=0.9±0.2y, 30.2±8.5 times/d  -Juul: ≥1 times/d, D=0.8±0.1y, 25.3±6.4 times/d  -NS: never any tobacco product  Dual users were excluded | Self-rated oral symptoms  Clinical periodontal status: CAL, PI, PD, MT, BOP, MBL  Outcome:  -Bad breath, pain in teeth and gums: more frequent in G1 compared to G2, G3 and G4  -PI, PD: sign. increased in G1 compared to G2, G3 and G4  -MT, BOP, CAL, MBL: not sign. different between groups | -CSS  -Small groups  -No verification of EC or Juul only use  -Short duration of EC and Juul use |
| 56 | | Xu et al., 2022 (45) | EC  +: CC  -: NS | LS: BL, FU at 6 months  101 Patients (≥21y) with periodontitis (completed the study):  -G1: 31 CC  -G2: 32 EC  -G3: 38 NS  (No age and gender distribution reported) | T/N History: Q? , self-reports?  (No selection criteria and values provided) | In saliva: oral microbiome and cytokines (IFN-γ, IL-1ß, IL-2, IL-4, IL-6, IL-8, IL-10, IL-12p70, IL-13, TNF-α)  Outcome:  -Severity of periodontitis at BL: G1 > G2 > G3  -EC use alters the oral microbiome  -EC may have similar potential as CC towards the bacterial composition over time  -Disease-associated pathogens are increased by EC use  -EC use was associated with increase of pro-inflammatory cytokines IFN-γ, IL-1ß, TNF-α (partly also in G1 and G2) | -Incomplete data on groups and T/N habits  -Small groups  -Dual use not considered  -EC only use not verified |
| 57 | Ye et al., 2020 (46) | | EC  +: CC, Dual  -: NU | CSS: 48 Subjects  -G1: 12 NU, M/F=2/10, 35.7±12.5y  -G2: 12 CC, M/F=5/7, 40.3±16.0y  -G3: 12 EC, M/F=10/2, 34.9±11.5y  -G4: 12 Dual, M/F=7/5, 39.4±11.8y | T/N History: Q, self-reports  Cotinine in saliva:  -NS: 0.6±0.6 ng/mL  -CC: 143±174 ng/mL  -EC: 180±273 ng/mL  -Dual: 299±433 ng/mL | BMs in saliva and GCF: inflammation, ox. stress, injury, repair, growth factors  Outcome: G1 / G2 / G3 / G4 (means)  -Saliva  --IL-1ß (pg/mL): 285 / 351 / 182 / 168  --PGE2 (pg/mL): 332^a^ / 589^a,b,c^ / 344^b^ /362^c^  -GCF: only BMs showing sign. differences, unit: pg/µg protein:  --EN-RAGE: 54^a^ / 116 / 80 / 156^a^  --RAGE: 16 / 7.4 / 2.2^a^ / 31^a^  --MMP-9: 95^a^ / 48 / 38^a^ / 84  --MPO: 216^a,b^ / 70^a^ / 40^b,c^ / 151^c^  ^a,b,c^: same letter means sign. diff. between groups | -CSS  -No criteria for groups provided  -EC only not verified  -Small group sizes  -Larger studies and LS are needed (authors) |
| 58 | | Guo et al., 2021 (47) | EC  +: CC  -: NS | LS: 95 Healthy subjects, >18y, 6 months FU with monthly visits  -G1: 353 NS  -G2: 70 CC  -G3: 30 EC | T/N History: Q, self-reports  + COex, in urine: cotinine, CEMA NNAL  -CC: ≥5 cig/d, ≥4 d/week in past year, >8 ppm COex  -EC: no CC in past ≥12 weeks, EC for ≥3 past months on ≥4 d/week, <6 ppm COex  -NS: <100 cig/lifetime, <6 ppm COex | AP sites in oral cells  Outcome: NS (N=26) / CC (N=27) / EC (N=18)  -AP sites/10^7^ nts (medians): 6.0^a^ / 5.7^b^ / 3.3^a,b^  *In vitro* experiments: PG may inhibit inflammation by bacteria in oral cavity, thus resulting in less AP sites (authors) | -Strengths: BOEs applied for verification of product use  -Small group sizes  -dGuo lower in NS than CC and EC  -CC older than NS and EC |
| 59 | | Bustamante et al., 2018 (48) | EC  +: CC  -: NS | CSS:  -G1: 20 EC, M/F=12/8, 31.3±12.2y  -G2: 20 CC, M/F=12/8, 41.9±11.4y  -G3: 19 NS, M/F=5/14, 40.6±17.2y | T/N History: Q, self-reports  BOEs in urine: N, NN, NNN  -EC: D=12.2±8.5y, start age CC: 15.9 ±3.1, 18.7 ±8.9 cig/d  -CC: start age: 14.7±3.6y, 15.9 ±4.0 cig/d | NNN in saliva:  -EC: 14.6±23.1 pg/mL  -CC: 94.5±176 pg/mL  -NS: 0.25±0.28 pg/mL  Results suggest endogenous formation of NNN in EC group | -CSS  -No BOE for combustion products included  -Small groups  -High variation of NNN in saliva (factors unknown) |
| 60 | | Pop et al., 2021 (49) | EC  +: CC  -: NS | CSS: 68 Participants  -G1: 25 CC, M/F=14/11, 22.4±1.4y  -G2: 23 EC, M/F=15/8, 21.5±1.7y  -G3: 20 NS, M/F=9/11, 21.7±1.6y | T/N History: Q, self-reports  -CC: ≥10 cig/d in last 12 months  -EC: daily use in last 12 months, no CC in last 3 months  -NS: no history of CC use  Dual users excluded | MN in oral exfoliated cells  Outcome: MN/1000 cells:  -CC: 3.6±1.08^a^  -EC: 3.21±1.12^b^  -NS: 1.95±1.05^a,b^  ^a,b^: same letter means sign. diff. between groups (p<0.01) | -CSS  -No verification of EC only use  -Small group sizes |
| 61 | | Pandaratho-diyil et al., 2021 (50) | EC  +: CC  -: NU | CSS: 88 Subjects  -G1: 30 NU, M/F=14/16, 31.6±13.5y  -G2: 29 CC, M/F=24/5, 32.3±12.8y  -G3: 29 EC, M/F=22/7, 26.0±7.4y | T/N History: Q, self-reports  -NU: no use of CC or EC  -CC: ≥6 months CC use, D=10.8±10.2y  -EC: ≥6 months EC use, D=2.2 ±1.9y  Dual use not considered | Salivary LDH activity (mU/mL):  -NU: 21.45±15.30^a,b^  -CC: 30.82±20.73^a^  -EC: 35.15±24.34^b^  ^a,b^: same letter means sign. diff. between groups (p<0.05) | -CSS  -No verification of EC only use  -Small group sizes  -Short EC use duration |
| 62 | | Ghazali et al., 2019 (51) | EC  +: CC  -: NS | LS: 135 Participants, BL and 6 months FU  -G1: 45 NS, M/F=16/29, 29.8±9.7y  -G2: 45 CC, M/F=44/1, 30.3±8.3y  -G3: 45 EC, M/F=22/7, 22.9±2.9y | T/N History: Q?, self-reports?  -CC: D ranges numbers: 1-10y/11-25y = 29/16  -EC: D ranges and numbers: 1-10y/11-25y = 45/0 | Dental caries score (DMFT):  No sign. differences between groups at BL and 6 months FU; sign. increase in all groups from BL to FU  Authors conclusions: EC use has detrimental effects on caries (?) | -No information how T/N history was assessed  -Results do not support conclusions |
| 63 | | Scherer et al. , 2022 (52) | EC, HTP  +: CC  -: NU | LS: BL, FU: Day 1, 2, 3  -G1: 10 NU, M/F=6/4, 32.9 (21-47) y  -G2: 10 CC, M/F=6/4, 35.2 (27-53) y  -G3: 10 EC, M/F=6/4, 36.1 (24-61) y  -G4: 10 HTP, M/F=6/4, 35.3 (22-56) y | T/N History: Q, self-reports  Verification of NCP use by BOEs  Consumption on Day 3 (controlled recording):  -CC: 11.8 cig/d  -EC: 156 puffs/d  -HTP; 16.5 sticks/d | NNN in saliva on Day 3::  G2^a,b^ >> G4^c^ > G3^b^ ≈ G1^a,c^  ^a,b,c^: same letter means sign. diff. between groups (p<0.05)  No evaluation of BL to FU changes  In G4: Sign. corr between NNN in saliva and thiocyanate in plasma (a catalyst of endogenous nitrosation) | -Strength: NCP use verified  -Small group sizes  -Short FU period |
| Footnotes:  ^1^: Internal ID number for the reviewed publications  ^2^: Groups with the same superscript letter (^a,b,c,…^) are reported to be statistically different from each other (p < 0.05 or better) | | | | | | | |

References

1. Akinkugbe, A.A.: Cigarettes, E-Cigarettes, and Adolescents' Oral Health: Findings from the Population Assessment of Tobacco and Health (Path) Study; JDR clinical and translational research (2018) 2380084418806870. DOI: 10.1177/2380084418806870

2. Akram, Z., S. Aati, A. Alrahlah, F. Vohra, and A. Fawzy: Longitudinal Evaluation of Clinical, Spectral and Tissue Degradation Biomarkers in Progression of Periodontitis among Cigarette and Electronic Cigarette Smokers; J Dent (2021) 103678. DOI: <https://doi.org/10.1016/j.jdent.2021.103678>

3. Al-Aali, K.A., M. Alrabiah, A.S. ArRejaie, T. Abduljabbar, F. Vohra, and Z. Akram: Peri-Implant Parameters, Tumor Necrosis Factor-Alpha, and Interleukin-1 Beta Levels in Vaping Individuals; Clin Implant Dent Relat Res 20 (2018) 410-415. DOI: <https://doi.org/10.1111/cid.12597>

4. Al-Hamoudi, N., A. Alsahhaf, M. Al Deeb, M. Alrabiah, F. Vohra, and T. Abduljabbar: Effect of Scaling and Root Planing on the Expression of Anti-Inflammatory Cytokines (Il-4, Il-9, Il-10, and Il-13) in the Gingival Crevicular Fluid of Electronic Cigarette Users and Non-Smokers with Moderate Chronic Periodontitis; Journal of periodontal & implant science 50 (2020) 74-82. DOI: 10.5051/jpis.2020.50.2.74

5. Alazmi, S.O., F.J. Almutairi, and B.A. Alresheedi: Comparison of Peri-Implant Clinicoradiographic Parameters among Non-Smokers and Individuals Using Electronic Nicotine Delivery Systems at 8 Years of Follow-Up; Oral health & preventive dentistry 19 (2021) 511-516. DOI: 10.3290/j.ohpd.b2082123

6. ALHarthi, S.S., M. BinShabaib, Z. Akram, I. Rahman, G.E. Romanos, and F. Javed: Impact of Cigarette Smoking and Vaping on the Outcome of Full-Mouth Ultrasonic Scaling among Patients with Gingival Inflammation: A Prospective Study; Clinical oral investigations 23 (2019) 2751-2758. DOI: <https://doi.org/10.1007/s00784-018-2725-2>

7. Ali, D., I. Kuyunov, J.K. Baskaradoss, and T. Mikami: Comparison of Periodontal Status and Salivary Il-15 and -18 Levels in Cigarette-Smokers and Individuals Using Electronic Nicotine Delivery Systems; BMC oral health 22 (2022) 655. DOI: 10.1186/s12903-022-02700-6

8. Alizadehgharib, S., A. Lehrkinder, A. Alshabeeb, A.-K. Östberg, and P. Lingström: The Effect of a Non-Tobacco-Based Nicotine Pouch on Mucosal Lesions Caused by Swedish Smokeless Tobacco (Snus); European journal of oral sciences 130 (2022).

9. AlJasser, R., M. Zahid, M. AlSarhan, D. AlOtaibi, and S. AlOraini: The Effect of Conventional Versus Electronic Cigarette Use on Treatment Outcomes of Peri-Implant Disease; BMC oral health 21 (2021) 480. DOI: 10.1186/s12903-021-01784-w

10. Alqahtani, F., M. Alqahtani, A.H. Albaqawi, A.A. Al-Kheraif, and F. Javed: Comparison of Cotinine Levels in the Peri-Implant Sulcular Fluid among Cigarette and Waterpipe Smokers, Electronic-Cigarette Users, and Nonsmokers; Clin Implant Dent Relat Res 21 (2019) 702-707. DOI: 10.1111/cid.12813

11. AlQahtani, M.A., A.S. Alayad, A. Alshihri, F.O.B. Correa, and Z. Akram: Clinical Peri-Implant Parameters and Inflammatory Cytokine Profile among Smokers of Cigarette, E-Cigarette, and Waterpipe; Clin Implant Dent Relat Res 13 (2018) 55-58. DOI: 10.1111/cid.12664

12. ArRejaie, A.S., K.A. Al-Aali, M. Alrabiah, F. Vohra, S.A. Mokeem, G.B. Rcdc, A. Alrahla, and T. Abduljabbar: Proinflammatory Cytokine Levels and Peri-Implant Parameters among Cigarette Smokers, Individuals Vaping Electronic Cigarettes and Non-Smokers; Journal of periodontology 90 (2019) 367-374. DOI: 10.1002/jper.18-0045

13. Atuegwu, N.C., M.F. Perez, C. Oncken, S. Thacker, E.L. Mead, and E.M. Mortensen: Association between Regular Electronic Nicotine Product Use and Self-Reported Periodontal Disease Status: Population Assessment of Tobacco and Health Survey; International journal of environmental research and public health 16 (2019). DOI: 10.3390/ijerph16071263

14. Bardellini, E., F. Amadori, G. Conti, and A. Majorana: Oral Mucosal Lesions in Electronic Cigarettes Consumers Versus Former Smokers; Acta odontologica Scandinavica (2018) 226-228. DOI: 10.1080/00016357.2017.1406613

15. BinShabaib, M., S.S. ALHarthi, Z. Akram, J. Khan, I. Rahman, G.E. Romanos, and F. Javed: Clinical Periodontal Status and Gingival Crevicular Fluid Cytokine Profile among Cigarette-Smokers, Electronic-Cigarette Users and Never-Smokers; Archives of oral biology 102 (2019) 212-217. DOI: <https://doi.org/10.1016/j.archoralbio.2019.05.001>

16. Cheng, G., J. Guo, S.G. Carmella, B. Lindgren, J. Ikuemonisan, J. Jensen, D.K. Hatsukami, S. Balbo, and S.S. Hecht: Increased Acrolein-DNA Adducts in Buccal Brushings of E-Cigarette Users; Carcinogenesis 43 (2022) 437-444. DOI: 10.1093/carcin/bgac026

17. Cho, J.H.: The Association between Electronic-Cigarette Use and Self-Reported Oral Symptoms Including Cracked or Broken Teeth and Tongue and/or inside-Cheek Pain among Adolescents: A Cross-Sectional Study; PLoS One 12 (2017) e0180506. DOI: 10.1371/journal.pone.0180506

18. Chopyk, J., C.M. Bojanowski, J. Shin, A. Moshensky, A.L. Fuentes, S.S. Bonde, D. Chuki, D.T. Pride, and L.E. Crotty Alexander: Compositional Differences in the Oral Microbiome of E-Cigarette Users; Frontiers in microbiology 12 (2021) 599664. DOI: 10.3389/fmicb.2021.599664

19. Stewart, C.J., T.A. Auchtung, N.J. Ajami, K. Velasquez, D.P. Smith, G.I. De La, R., R. Salas, and J.F. Petrosino: Effects of Tobacco Smoke and Electronic Cigarette Vapor Exposure on the Oral and Gut Microbiota in Humans

a Pilot Study; PeerJ, Vol 6, p e 4693 (2018).

20. Cichonska, D., A. Kusiak, B. Kochanska, J. Ochocinska, and D. Swietlik: Influence of Electronic Cigarettes on Selected Antibacterial Properties of Saliva; International journal of environmental research and public health 16 (2019). DOI: 10.3390/ijerph16224433

21. Cichońska, D., A. Kusiak, B. Kochańska, J. Ochocińska, and D. Świetlik: Influence of Electronic Cigarettes on Selected Physicochemical Properties of Saliva; International journal of environmental research and public health 19 (2022). DOI: 10.3390/ijerph19063314

22. Faridoun, A., A.S. Sultan, M. Ann Jabra-Rizk, and T.F. Meiller: Salivary Biomarker Profiles in E-Cigarette Users and Conventional Smokers: A Cross-Sectional Study; Oral Dis 27 (2021) 277-279. DOI: 10.1111/odi.13533

23. Franco, T., S. Trapasso, L. Puzzo, and E. Allegra: Electronic Cigarette: Role in the Primary Prevention of Oral Cavity Cancer; Clinical medicine insights. Ear, nose and throat 9 (2016) 7-12. DOI: 10.4137/cment.s40364

24. Ganesan, S.M., S.M. Dabdoub, H.N. Nagaraja, M.L. Scott, S. Pamulapati, M.L. Berman, P.G. Shields, M.E. Wewers, and P.S. Kumar: Adverse Effects of Electronic Cigarettes on the Disease-Naive Oral Microbiome; Sci Adv 6 (2020) eaaz0108. DOI: 10.1126/sciadv.aaz0108

25. Holliday, R., P.M. Preshaw, V. Ryan, F.F. Sniehotta, S. McDonald, L. Bauld, and E. McColl: A Feasibility Study with Embedded Pilot Randomised Controlled Trial and Process Evaluation of Electronic Cigarettes for Smoking Cessation in Patients with Periodontitis; Pilot and feasibility studies 5 (2019) 74. DOI: 10.1186/s40814-019-0451-4

26. Huilgol, P., S.P. Bhatt, N. Biligowda, N.C. Wright, and J.M. Wells: Association of E-Cigarette Use with Oral Health: A Population-Based Cross-Sectional Questionnaire Study; Journal of public health (Oxford, England) 41 (2019) 354-361. DOI: 10.1093/pubmed/fdy082

27. Ibraheem, W.I., H.I. Fageeh, R.S. Preethanath, F.A. Alzahrani, A.S. Al-Zawawi, D.D. Divakar, and A.A. Al-Kheraif: Comparison of Rankl and Osteoprotegerin Levels in the Gingival Crevicular Fluid of Young Cigarette- and Waterpipe-Smokers and Individuals Using Electronic Nicotine Delivery Systems; Archives of oral biology 115 (2020). DOI: 10.1016/j.archoralbio.2020.104714

28. Irusa, K.F., M. Finkelman, B. Magnuson, T. Donovan, and S.E. Eisen: A Comparison of the Caries Risk between Patients Who Use Vapes or Electronic Cigarettes and Those Who Do Not: A Cross-Sectional Study; Journal of the American Dental Association (1939) 153 (2022) 1179-1183. DOI: 10.1016/j.adaj.2022.09.013

29. Javed, F., T. Abduljabbar, F. Vohra, H. Malmstrom, I. Rahman, and G.E. Romanos: Comparison of Periodontal Parameters and Self-Perceived Oral Symptoms among Cigarette Smokers, Individuals Vaping Electronic Cigarettes, and Never-Smokers; Journal of periodontology 88 (2017) 1059-1065. DOI: 10.1902/jop.2017.170197

30. Jeong, W., D.W. Choi, Y.K. Kim, H.J. Lee, S.A. Lee, E.C. Park, and S.I. Jang: Associations of Electronic and Conventional Cigarette Use with Periodontal Disease in South Korean Adults; Journal of periodontology 91 (2020) 55-64. DOI: 10.1002/jper.19-0060

31. Karaaslan, F., A. Dikilita, and U. Yi_it: The Effects of Vaping Electronic Cigarettes on Periodontitis; Australian dental journal 65 (2020) 143-149.

32. Miluna, S., R. Melderis, L. Briuka, I. Skadins, R. Broks, J. Kroica, and D. Rostoka: The Correlation of Swedish Snus, Nicotine Pouches and Other Tobacco Products with Oral Mucosal Health and Salivary Biomarkers; Dentistry journal 10 (2022). DOI: 10.3390/dj10080154

33. Mokeem, S.A., M.N. Alasqah, D. Michelogiannakis, A.A. Al-Kheraif, G.E. Romanos, and F. Javed: Clinical and Radiographic Periodontal Status and Whole Salivary Cotinine, Il-1beta and Il-6 Levels in Cigarette- and Waterpipe-Smokers and E-Cig Users; Environmental toxicology and pharmacology 61 (2018) 38-43. DOI: 10.1016/j.etap.2018.05.016

34. Park, B., H. Koh, M. Patatanian, H. Reyes-Caballero, N. Zhao, J. Meinert, J.T. Holbrook, L.I. Leinbach, and S. Biswal: The Mediating Roles of the Oral Microbiome in Saliva and Subgingival Sites between E-Cigarette Smoking and Gingival Inflammation; BMC microbiology 23 (2023). DOI: 10.1186/s12866-023-02779-z

35. Pushalkar, S., B. Paul, Q. Li, J. Yang, R. Vasconcelos, S. Makwana, J.M. Gonzalez, S. Shah, C. Xie, M.N. Janal, E. Queiroz, M. Bederoff, J. Leinwand, J. Solarewicz, F. Xu, E. Aboseria, Y. Guo, D. Aguallo, C. Gomez, A. Kamer*, et al.*: Electronic Cigarette Aerosol Modulates the Oral Microbiome and Increases Risk of Infection; iScience 23 (2020). DOI: 10.1016/j.isci.2020.100884

36. Reeve, G.S., M.R. Rostami, R.F. Reich, D.A. Behrman, P.L. Leopold, R.G. Crystal, and Y. Strulovici-Barel: Oral Epithelium Response of Electronic Cigarette Users to Electronic Cigarette; Journal of oral pathology & medicine : official publication of the International Association of Oral Pathologists and the American Academy of Oral Pathology 52 (2023) 431-439. DOI: 10.1111/jop.13380

37. Schwarzmeier LÂ, T., B.S. da Cruz, C.C.P. Ferreira, B. Carvalho, M.G.O. Alves, C.F. Lima Carta, J.R. Scholz, and J.D. Almeida: E-Cig Might Cause Cell Damage of Oral Mucosa; Oral surgery, oral medicine, oral pathology and oral radiology 131 (2021) 435-443. DOI: 10.1016/j.oooo.2020.11.009

38. Tatullo, M., S. Gentile, F. Paduano, L. Santacroce, and M. Marrelli: Crosstalk between Oral and General Health Status in E-Smokers; Medicine (Baltimore) 95 (2016) e5589. DOI: 10.1097/md.0000000000005589

39. Thomas, S.C., F. Xu, S. Pushalkar, Z. Lin, N. Thakor, M. Vardhan, Z. Flaminio, A. Khodadadi-Jamayran, R. Vasconcelos, A. Akapo, E. Queiroz, M. Bederoff, M.N. Janal, Y. Guo, D. Aguallo, T. Gordon, P.M. Corby, A.R. Kamer, X. Li, and D. Saxena: Electronic Cigarette Use Promotes a Unique Periodontal Microbiome; mBio 13 (2022). DOI: 10.1128/mbio.00075-22

40. Tommasi, S., H. Blumenfeld, and A. Besaratinia: Vaping Dose, Device Type, and E-Liquid Flavor Are Determinants of DNA Damage in Electronic Cigarette Users; Nicotine & tobacco research : official journal of the Society for Research on Nicotine and Tobacco 25 (2023) 1145-1154. DOI: 10.1093/ntr/ntad003

41. Tommasi, S., A.W. Caliri, A. Caceres, D.E. Moreno, M. Li, Y. Chen, K.D. Siegmund, and A. Besaratinia: Deregulation of Biologically Significant Genes and Associated Molecular Pathways in the Oral Epithelium of Electronic Cigarette Users; International journal of molecular sciences 20 (2019). DOI: 10.3390/ijms20030738

42. Vemulapalli, A., S.R. Mandapati, A. Kotha, and S. Aryal: Association between Vaping and Untreated Caries: A Cross-Sectional Study of National Health and Nutrition Examination Survey 2017-2018 Data; Journal of the American Dental Association (1939) 152 (2021) 720-729. DOI: 10.1016/j.adaj.2021.04.014

43. Verma, A., K. Anand, M. Bhargava, A. Kolluri, M. Kumar, and D.H. Palve: Comparative Evaluation of Salivary Biomarker Levels in E-Cigarette Smokers and Conventional Smokers; Journal of pharmacy & bioallied sciences 13 (2021) S1642-s1645. DOI: 10.4103/jpbs.jpbs_393_21

44. Vohra, F., I.A. Bukhari, S.A. Sheikh, R. Albaijan, and M. Naseem: Comparison of Self-Rated Oral Symptoms and Periodontal Status among Cigarette Smokers and Individuals Using Electronic Nicotine Delivery Systems; Journal of American college health : J of ACH 68 (2020) 788-793. DOI: 10.1080/07448481.2019.1709476

45. Xu, F., S. Pushalkar, Z. Lin, S.C. Thomas, J.K. Persaud, M.A. Sierra, M. Vardhan, R. Vasconcelos, A. Akapo, Y. Guo, T. Gordon, P.M. Corby, A.R. Kamer, X. Li, and D. Saxena: Electronic Cigarette Use Enriches Periodontal Pathogens; Mol Oral Microbiol (2022). DOI: 10.1111/omi.12361

46. Ye, D., S. Gajendra, G. Lawyer, N. Jadeja, D. Pishey, S. Pathagunti, J. Lyons, P. Veazie, G. Watson, S. McIntosh, and I. Rahman: Inflammatory Biomarkers and Growth Factors in Saliva and Gingival Crevicular Fluid of E-Cigarette Users, Cigarette Smokers, and Dual Smokers: A Pilot Study; Journal of periodontology 91 (2020) 1274-1283. DOI: 10.1002/jper.19-0457

47. Guo, J., J. Ikuemonisan, D.K. Hatsukami, and S.S. Hecht: Liquid Chromatography-Nanoelectrospray Ionization-High-Resolution Tandem Mass Spectrometry Analysis of Apurinic/Apyrimidinic Sites in Oral Cell DNA of Cigarette Smokers, E-Cigarette Users, and Nonsmokers; Chem Res Toxicol (2021). DOI: 10.1021/acs.chemrestox.1c00308

48. Bustamante, G., B. Ma, G. Yakovlev, K. Yershova, C. Le, J. Jensen, D.K. Hatsukami, and I. Stepanov: Presence of the Carcinogen *N*'-Nitrosonornicotine in Saliva of E-Cigarette Users; Chem. Res. Toxicol. 31 (2018) 731-738. DOI: 10.1021/acs.chemrestox.8b00089

49. Pop, A.M., R. Coroș, A.M. Stoica, and M. Monea: Early Diagnosis of Oral Mucosal Alterations in Smokers and E-Cigarette Users Based on Micronuclei Count: A Cross-Sectional Study among Dental Students; International journal of environmental research and public health 18 (2021) 13246.

50. Pandarathodiyil, A.K., A. Ramanathan, R. Garg, J.G. Doss, F.B. Abd Rahman, W.M.N. Ghani, and S. Warnakulasuriya: Lactate Dehydrogenase Levels in the Saliva of Cigarette and E-Cigarette Smokers (Vapers): A Comparative Analysis; Asian Pacific journal of cancer prevention : APJCP 22 (2021) 3227-3235. DOI: 10.31557/apjcp.2021.22.10.3227

51. Ghazali, A.F., A.F. Ismail, and A. Daud: Caries Experience among Cigarette and E-Cigarette Users: A 6-Month Prospective Study; Journal of Pharmaceutical Sciences and Research 11 (2019) 2566-2569.

52. Scherer, G., M. Scherer, J. Mutze, T. Hauke, and N. Pluym: Assessment of the Exposure to Tobacco-Specific Nitrosamines and Minor Tobacco Alkaloids in Users of Various Tobacco/Nicotine Products; Chem Res Toxicol 35 (2022) 684-693. DOI: 10.1021/acs.chemrestox.2c00020
